# Pharmacogenomics characterization of the MDM2 inhibitor MI-773 reveals candidate tumours and predictive biomarkers

**DOI:** 10.1038/s41698-021-00235-7

**Published:** 2021-10-28

**Authors:** Vincent Vuaroqueaux, Hans R. Hendriks, Hoor Al-Hasani, Anne-Lise Peille, Samayita Das, Heinz-Herbert Fiebig

**Affiliations:** 1 4HF Biotec GmbH, Am Flughafen 14, 79108 Freiburg, Germany; 2 Hendriks Pharmaceutical Consulting, 1443 LR Purmerend, The Netherlands

**Keywords:** Computational biology and bioinformatics, Molecular medicine, Targeted therapies, Predictive markers

## Abstract

MI-773 is a recently developed small-molecule inhibitor of the mouse double minute 2 (MDM2) proto-oncogene. Preclinical data on the anti-tumour activity of MI-773 are limited and indicate that tumour cell lines (CLs) with mutated *TP53* are more resistant to MI-773 than wild type *TP53*. Here, we explored the compound’s therapeutic potential in vitro using a panel of 274 annotated CLs derived from a diversity of tumours. MI-773 exhibited a pronounced selectivity and moderate potency, with anti-tumour activity in the sub-micromolar range in about 15% of the CLs. The most sensitive tumour types were melanoma, sarcoma, renal and gastric cancers, leukaemia, and lymphoma. A COMPARE analysis showed that the profile of MI-773 was similar to that of Nutlin-3a, the first potent inhibitor of p53–MDM2 interactions, and, in addition, had a superior potency. In contrast, it poorly correlates with profiles of compounds targeting the p53 pathway with another mechanism of action. OMICS analyses confirmed that MI-773 was primarily active in CLs with wild type *TP53*. In silico biomarker investigations revealed that the *TP53* mutation status plus the aggregated expression levels of 11 genes involved in the p53 signalling pathway predicted sensitivity or resistance of CLs to inhibitors of p53–MDM2 interactions reliably. The results obtained for MI-773 could help to refine the selection of cancer patients for therapy.

## Introduction

The tumour protein p53 is a transcription factor involved in cell cycle regulation, apoptotic cell death, and maintenance of genetic stability^1,2^. The *TP53* gene, encoding for p53, is mutated in ~40% of human cancers, the mutation frequency differs between tumour types and ranges from <5% to >90%^3,4^. *TP53* mutations profoundly affect tumour cell genomic structure, expression, and clinical outlook. Loss of p53 integrity is correlated with poor patient survival for multiple tumour types^5^. Mouse double minute 2 (MDM2) primarily regulates the expression of p53, which makes it an attractive target for cancer therapy^6^.

MDM2 is a ubiquitin ligase that facilitates p53 proteasomal degradation. Upon stress such as DNA damage, the protein–protein interaction between p53 and MDM2 is disrupted, resulting in elevated p53 levels, cell cycle arrest, DNA repair, or elimination by apoptosis^7–9^. *MDM2* amplification and overexpression are found at a high frequency in soft tissue tumours, e.g., liposarcoma, at a lower frequency in glioblastoma and breast cancer, but not in tumour types like leukaemia, lymphoma, and melanoma. *MDM2* overexpression leads to the downregulation of p53 and, consequently, loss of apoptotic function and cell cycle arrest in wild type *TP53* tumours and is associated with drug resistance to chemotherapeutics such as cisplatin^10–13^. Small molecule inhibitors which occupy the p53-binding pocket of MDM2 disrupt the MDM2–p53 interaction, leading to the stabilisation of p53 and activation of the pathway^14^. Several MDM2 inhibitors are currently in clinical development^15^.

The MDM2 inhibitor MI-773 (SAR405838, MI-77301) is a recent small molecule that binds to MDM2 with high affinity (*K*_i_ = 0.88 nM)^16^. It is active in wild type *TP53* cell lines (CLs) from leukaemia and solid tumours in vitro and induces either durable tumour regression or effective tumour growth inhibition in subcutaneously transplanted patient-derived tumour models (PDX). The compound is less efficacious in CLs and PDX with mutated *TP53*^16–19^. Two-Phase I studies were conducted in patients with locally advanced or metastatic solid tumours (mainly wild type *TP53*) either as single-agent or in combination with the oral MEK1+2 inhibitor pimasertib. In both studies, MI-773 showed an acceptable safety profile^20,21^.

Preclinical and early clinical proof-of-concept studies have shown that small-molecule MDM2 inhibitors block the p53–MDM2 interaction, activate p53 in patients, and have acceptable safety profiles^9,22,23^. The published preclinical data on the anti-tumour activity of MI-773 are limited and concern investigations in a small panel of human leukaemia and solid tumour CLs (with either wild type, mutated or deleted *TP53*). Since ~60% of all human tumours are wild type *TP53*, and preclinical data suggest that CLs with mutated *TP53* are more resistant to MI-773 than wild type *TP53*^24^, it is of particular interest to explore the therapeutic potential of the compound in a large number of haematological and solid tumours with both wild type and mutated *TP53*. These data prompted us to explore the therapeutic potential of MI-773 in depth. Here, we report the results of this in vitro and pharmacogenomic study, revealing candidate tumours for treatment with MI-773 and a biomarker set for patient stratification.

## Results

### MI-773 exerts anti-tumour activity in vitro

**Fig. 1:**
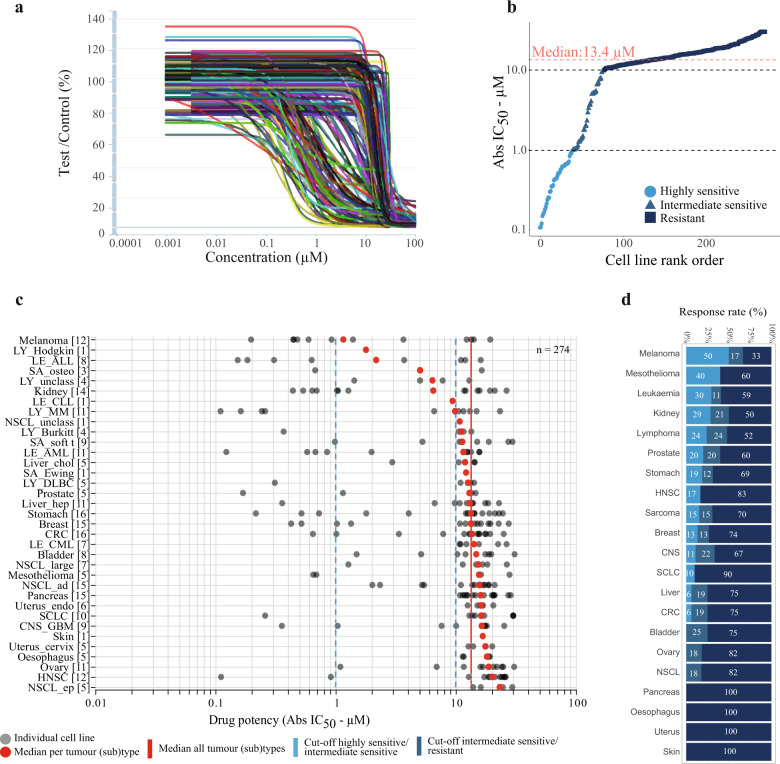
The anti-tumour activity of MI-773 in the 274-CL panel in vitro. **a** The individual dose–response curves of all CLs to MI-773. Each line corresponds to a fitted displacement curve for a CL. *x*-axis, MI-773 concentration in µM. *Y*-axis, drug effects on cell proliferation and survival expressed as Test/Control × 100 (%) values. **b** Rank ordered Abs IC_50_ values obtained for each CL. The dashed red line depicts the median Abs IC_50_ value. Dashed black lines are the cut-offs of 1 and 10 µM identifying highly sensitive (<1 µM), intermediate sensitive ([1, 10 [µM) and resistant (≥10 µM) CLs. **c** Scatter plot of the drug potency expressed with Abs IC_50_ value per CL (*x*-axis). On the *y*-axis, the histological (sub)types are sorted from top to bottom by increasing median Abs IC_50_ values. Between brackets the total number of CLs within a tumour (sub)type. *Abbreviations*: CNS_GBM central nervous system: glioblastoma, CRC colorectal cancer, HNSC head &neck squamous cell, LE_ALL acute lymphoblastic leukaemia, LE_AML acute myeloid leukaemia, LE_CLL chronic lymphocytic leukaemia, LE_CML chronic myelogenous leukaemia, Liver_chol Liver_cholangioma, Liver_hep liver_hepatocellular, LY_Burkitt lymphoma_Burkitt, LY_DLBC lymphoma_Diffuse large B cells, LY_Hodgkin lymphoma_Hodgkin, LY_MM lymphoma_multiple myeloma, LY_unclass lymphoma_unclassified, NSCL_ad non-small cell lung_adenocarcinoma, NSCL_ep non-small cell lung_epidermoid, NSCL_large non-small cell lung_large cells, NSCL_unclass non-small cell lung_unclassified, SA_Ewing Sarcoma Ewing, SA_osteo osteosarcoma, SA_soft t sarcoma soft tissue, SCLC small cell lung, uterus_endo uterus_endometrium. **d** The percentage of highly sensitive (light blue), intermediate sensitive (grey-blue) and resistant CLs (dark blue) to MI-773 across different tumour types.

Figure 1a shows the dose–response curves of the individual 274 CLs and the large variety in sensitivity to the compound. The absolute IC_50_ values (Abs IC_50_) of the CLs ranged from 0.11 to 30.8 µM (Supplementary Table 1) with a median Abs IC_50_ of 13.4 µM (IQR: 5.7–17.6 µM). Three distinct patterns of sensitivity appeared in the rank order of the Abs IC_50_ values (Fig. 1b). The first subset of 40 CLs highly sensitive to MI-773 (15%; Abs IC_50_ values <1 µM), the second subset of 38 intermediate sensitive CLs (14%; Abs IC_50_ [1, 10[ µM), and the third subset of 196 resistant CLs (71%; Abs IC_50_ values ≥ 10 µM). The subset of highly sensitive CLs was enriched in melanoma, mesothelioma, renal cancer, leukaemia, and lymphoma. The sensitivity of CLs within a given tumour type was highly variable (Fig. 1c, d). Melanoma, renal cancer, sarcoma, and gastric cancer were the solid tumour types with the most sensitive CLs (>10 CLs/tumour type and >30% highly and intermediate sensitive CLs), and acute myeloid leukaemia (AML) and multiple myeloma were the most sensitive haematological tumours (Fig. 1c, d, Supplementary Table 2).

### Validation of MI- 773’s mechanism of action

**Fig. 2:**
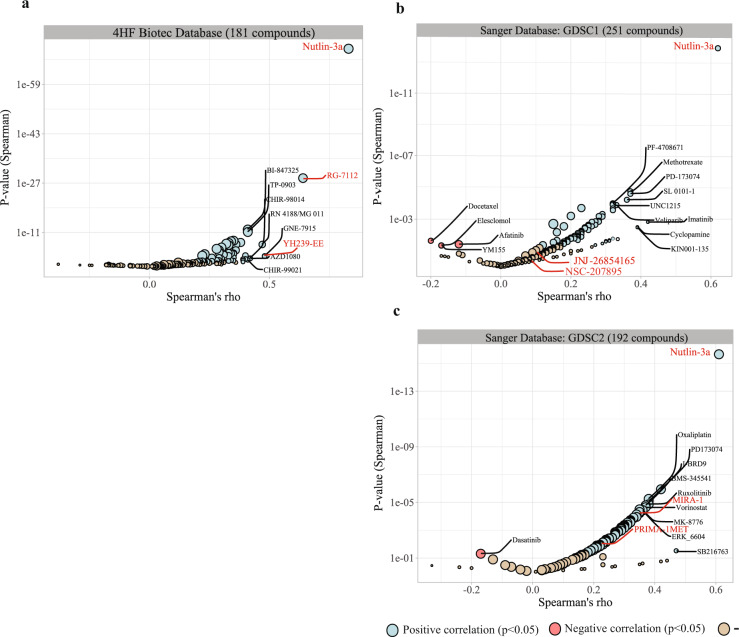
COMPARE analysis of MI-773 mechanism of action. Volcano plot showing the anti-cancer drugs correlated with MI-773 Abs IC_50_ (COMPARE analysis). *x*-axis: Rho values obtained (Spearman), *y*-axis: *p*-values. Blue: anti-cancer drugs with a positive correlation (*p*-values < 0.05), red: negative correlation (*p*-values < 0.05) (light brown: not significant). **a** 4HF Biotec database, **b** and **c** Sanger GDSC1 and GDSC2 databases. The size of the circles is proportional to the number of data points compared.

To validate the mechanism of action of MI-773, we performed a COMPARE analysis in which the MI-773 Abs IC_50_ values (4HF Biotec data) were correlated with the Abs IC_50_ values obtained from 181 standard anti-cancer agents with a known mechanism of action from our proprietary drug sensitivity database for cytotoxic potency and selectivity^25,26^. The agents most closely related to the MI-773 inhibitory profile were three inhibitors of the p53–MDM2 interaction: Nutlin-3a (*ϼ* = 0.83), RG-7112 (*ϼ* = 0.64), and YH239-EE (*ϼ* = 0.48) (Spearman correlation test, all *p*-values < 0.0005) (Fig. 2a, Supplementary Table 3a). Nutlin-3a, as the reference compound for MDM2 inhibition, was tested in 273 out of the 274-CL panel used for MI-773. Its median Abs IC_50_ value was 30 µM, [IQR: 12.8–33.1 µM], twice that of MI-773, and with a similar pattern of tumour (sub)type response as that of MI-773 (Supplementary Fig. 1a).

For independent validation of our results, the MI-773 Abs IC_50_ values were tested in a second and third COMPARE analysis against the GDSC1 and GDSC2 databases (443 drug sensitivity datasets, 987 CLs) from the COSMIC cancer cell line database^27^. Nutlin-3a showed also here the highest correlation with MI-773 (Spearman correlation test GDSC1, *ϼ* = 0.62, *p* = 1.1E^−14^; Spearman correlation test GDSC2, *ϼ* = 0.61, *p* < 2.2E^−16^), confirming MI-773’s mechanism of action (Fig. 2b, c, Supplementary Table 3b, c). Interestingly, the correlations with other inhibitors of the p53-signalling pathway with other modalities of action were more modest. The mutant p53-reactivating small molecules MIRA-1 and PRIMA-1MET showed a correlation coefficient of 0.35 (*p* = 5.94E^−05^) and 0.23 (*p* = 7.66E^−03^), respectively; the correlation coefficient of the inhibitor of MDM2–proteasome interaction JNJ-26854165 (Serdemetan) was 0.12 (*p* = 1.62E^−01^) and that of the MDMx inhibitor NSC-207895 was 0.09 (*p* = 3.04E^−01^).

The median Abs IC_50_ value of Nutlin-3a was lower in both GDSC1 (46.9 µM, IQR: 13.1–96.9 µM) and GDSC2 databases (125.96 µM, IQR: 35.02–296 µM) than in our in vitro test (Supplementary Fig. 1b, c). The drug response pattern observed across tumour types matched well with our MI-773 and Nutlin-3a internal data: leukaemia, lymphoma, melanoma, neuroblastoma, mesothelioma, and renal cancer were the most sensitive tumour types to MDM2 inhibition.

### Molecular features associated with the CL sensitivity to MI-773

To analyse molecular determinants of CL sensitivity to MI-773, whole-exome mutations, somatic copy number alterations (SCNA), and gene expression profiles were available for 237/274 CLs. This CL panel had similar characteristics as the 274-CL panel regarding (sub)types and distribution into the three subsets of MI-773 sensitivity (Supplementary Table 4).

**Fig. 3:**
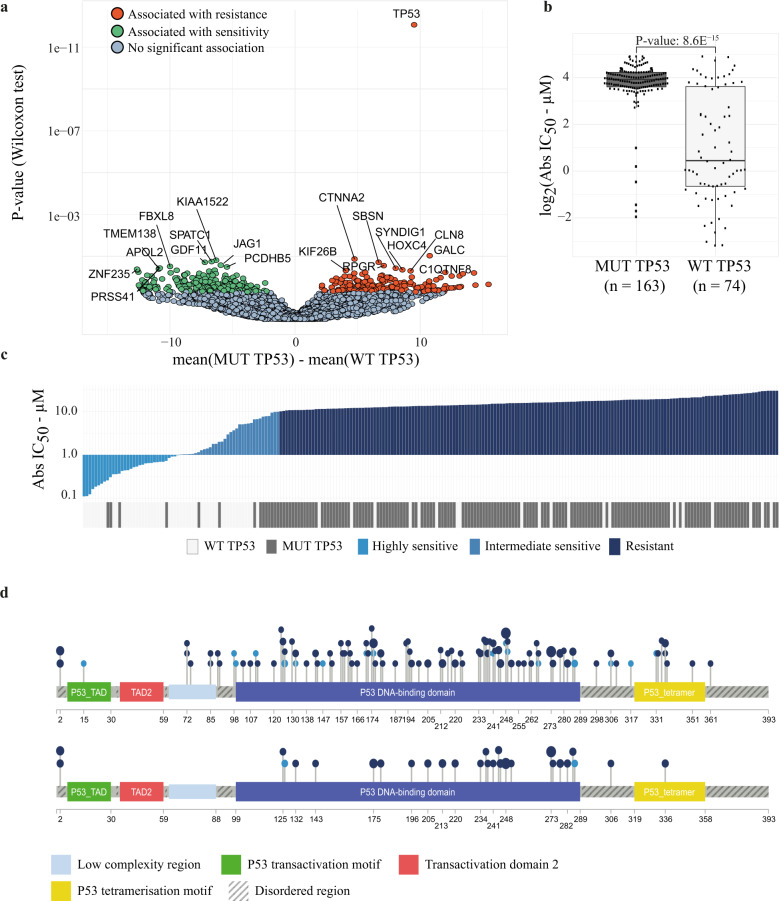
Genomic alterations associated with sensitivity toward MI-773. **a** Volcano plot showing the mutated genes positively or negatively associated with the MI-773 Abs IC_50_ values. *x*-axis, the difference between mean Abs IC_50_ in wild type vs. mutated groups, *y*-axis: *p*-values in log_10_ scale. Green dots: association with lower MI-773 Abs IC_50_, red dots: with higher Abs IC_50_ (two-sided Wilcoxon *p*-value < 0.05), grey dots: association not significant (*p*-value ≥ 0.05). **b** Boxplot of MI-773 Abs IC_50_ in the *TP53* wild type (WT *TP53*) and mutant (MUT *TP53*) groups. *p*-Value was obtained from the two-sided Wilcoxon test. Box limits are the 1st and 3rd interquartile; the inner horizontal line is the median, whiskers extend 1.5 times the interquartile range from the 1st and 3rd interquartile, data points were plotted as black dots. **c** The association between CL sensitivity to MI-773 and *TP53* status. The upper panel shows a waterfall plot of the MI-773 Abs IC_50_ of all 237 CLs. The lower panel shows the mutation status of the *TP53* gene in the corresponding CLs. **d** Lollipop plot showing the location on p53 protein domains of the *TP53* mutations identified on the 237-CL panel. Top: all *TP53* mutations, bottom: mutations retrieved in more than one model (recurrent). The colours of the circles reflect the response classes to the MI-773.

First, we determined genomic alterations associated with CL sensitivity and resistance to MI-773. Whole-exome sequencing data unveiled 17,196 mutated genes present in at least two CLs of our panel. Gene per gene testing revealed 535 mutated genes significantly associated with MI-773 Abs IC_50_ levels (two-sided Wilcoxon test, *p*-value < 0.05). *TP53* mutations were by far the major genetic determinants for resistance to MI-773 (two-sided Wilcoxon test, *p* = 8.56E^−15^) (Fig. 3a, b). When the p-values were adjusted using the Benjamini and Hochberg correction, *TP53* remained the only significant mutated gene out of the 535 (adjusted *p* = 1.47E^−10^). *TP53* mutations were present in 69% (163/237) of the CLs in our panel (Fig. 3b, Supplementary Table 5). The MI-773 median Abs IC_50_ value of the group of *TP53* mutated CLs was 11-fold higher (15.1 µM, IQR: 12.2–18.8 µM) than that of the wild type *TP53* CL group (1.4 µM, IQR: 0.6–12.3 µM) (Fig. 3b).

Figure 3c shows the landscape of the *TP53*-mutated CLs and the corresponding MI-773 Abs IC_50_ values. It highlights that 91% of CLs (149/163) with *TP53* mutations were strongly resistant to MI-773 (Abs IC_50_ ≥ 10 µM, Fisher´s exact test: *p*-value < 2.2E^−16^) (Supplementary Table 5). In-depth analysis of *TP53* mutation types (Fig. 3d upper lollipop plot) confirmed the diversity of alterations and localisations. *TP53* mutations were mainly present in the p53 DNA-binding domain, and a part of them was found in two or more CLs (Fig. 3d lower lollipop plot). In summary, the presence of a *TP53* mutation strongly indicated resistance to MI-773, irrespective of the mutation type or its location.

However, sensitivity to MI-773 did not only depend on the mutation status of *TP53*; 21 out of 74 CLs with wild type *TP53* were resistant to the compound, whereas 14 out of 163 *TP53*-mutated CLs were sensitive (Supplementary Table 5). We verified whether genomic alterations in *MDM2* contributed to MI-773 resistance in wild type *TP53* CLs. Only two out of the 21 wild types *TP53* CLs resistant to MI-773 showed *MDM2* mutations. High *MDM2* SCNA (PICNIC value ≥8) was absent in our 237-CL panel. In the overall CL panel, 135 CLs (57%) showed a moderate SCNA at *MDM2* loci (PICNIC value >2), which was, however, not associated with higher expression levels of *MDM2* and response to MI-773 (data not shown). Thus, resistance to MI-773 in wild type *TP53* CLs in our panel was not related to *MDM2* gene alterations. When we broadened the analysis to whole-exome mutations and SCNA of other genes present in these 21 wild type *TP53* CLs, we did not find any genomic alteration that could explain the resistance to MI-773.

**Fig. 4:**
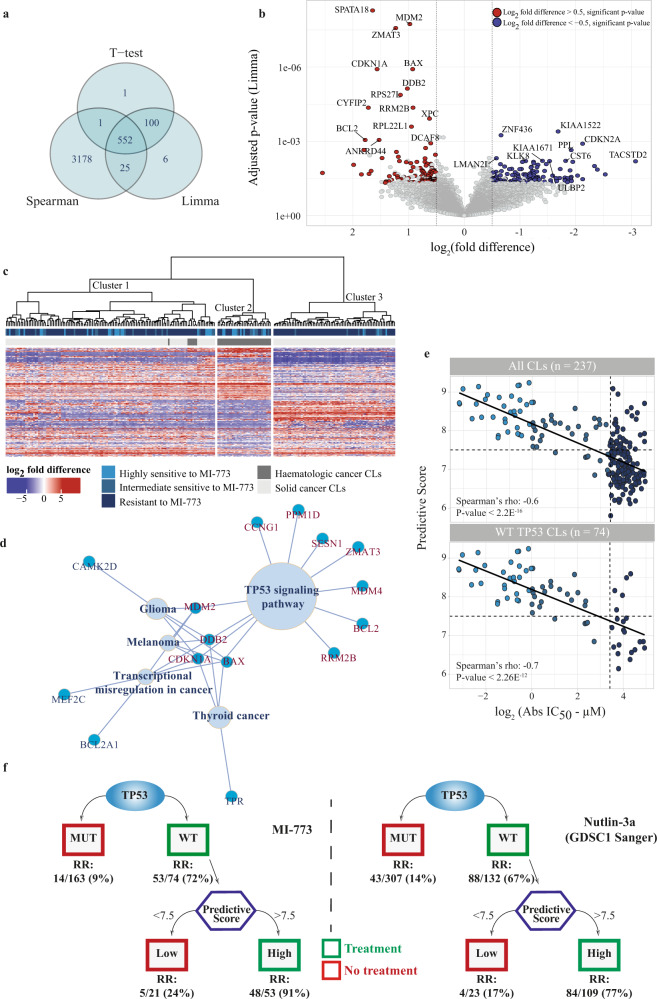
Association of gene expression with sensitivity toward MI-773. **a** Venn diagram showing the number of Affymetrix Human Genome U133 plus 2.0 probe sets significantly associated with MI-773 Abs IC_50_ according to the *t*-test, Limma and Spearman statistical tests (adjusted *p*-value < 0.05). **b** Volcano plot showing levels of significance of individual genes for the association of their expression levels with MI-773 Abs IC_50_ values. *x*-axis, log_2_ fold difference of gene expression level between MI-773 response groups; *y*-axis, Limma adjusted *p*-values on log_10_ scale. The red and blue dots show the genes having an expression significantly associated with the response to MI-773 (significant adjusted *p*-value in Limma, *t*-test and Spearman) and a log_2_ fold difference >0.5 (red dots) or <−0.5 (blue dots) as calculated by Limma. The grey dots depict the genes neither significantly associated with the response to MI-773 (adjusted *p*-value ≥ 0.05 in Limma, *t*-test and Spearman) nor having fold difference below 0.5 of amplitude. **c** Heatmap with unsupervised hierarchical clustering of 237 differentially expressed genes (fold difference > ±0.5). First upper bar shows response groups to MI-773, second upper bar shows the type of cancer cell lines (haematologic or solid). **d** KEGG enrichment analysis of the 113 genes whose high expression was positively associated with MI-773 sensitivity. **e** Spearman correlation analysis between MI-773 Abs IC_50_ values and the gene expression-based predictive score. Upper plot: all CLs, lower plot: wild type *TP53* CLs only. The solid line shows the correlation between the MI-773 Abs IC_50_ values and the gene expression-based predictive score. Dots are coloured according to their response groups to MI-773. **f** Decisional tree performance for the CL classification into groups of sensitive and resistant to MDM2 inhibition. RR: response rates. Left: MI-773, right: Nutlin-3a.

Next, we screened the transcriptome of the 237-CL panel to identify genes whose expression level was associated with the sensitivity to MI-773 (see the “Methods” section). After removing probe sets not showing consistent gene expression (probe sets with values below five in all samples were excluded), a total of 31,751 probe sets were tested for an association between transcript expression level and MI-773 Abs IC_50_ values. For a robust and stringent result, the analysis was carried out by applying three statistical tests (two-sided *t*-test, Limma (cut-off for MI-773 Abs IC_50_ at the 30th percentile: 10.7 μM), and Spearman correlation test). At the intersection of the three statistical tests, we obtained a total of 552 probe sets significantly associated with the MI-773 Abs IC_50_ (adjusted *p*-value < 0.05) (Fig. 4a). From these probe sets, we retained 316 (each corresponding to a unique gene) that had, according to the Jetset scoring method, the best specificity, coverage, and degradation resistance^28^. The volcano plot shown in Fig. 4b displays 113 out of 316 genes with high expression associated with sensitivity to MI-773 and 124 with higher expression in resistant CLs (genes with fold difference amplitude > ±0.5). The most significant genes with high expression associated with MI-773 sensitivity include *SPATA18*, *ZMAT3*, *CDKN2A*, *BAX*, and the target *MDM2*.

Kyoto Encyclopedia of Genes and Genomes (KEGG) and Gene Ontology (GO) biological process (BP) enrichment analyses were performed on the 113 genes with high expression in CLs sensitive to MI-773. The outcome confirmed that the genes were dominantly related to the compound’s mechanism of action with 11 genes associated with the p53-signalling pathway (Fig. 4d, Supplementary Table 6a). Three genes (*MDM2*, *MDM4*, *PPM1D*) have direct protein–protein interaction with *TP53*; others were involved in the cell cycle (*CDKN1A*, *ZMAT3*, *CCNG1*, *RRM2B*), apoptosis (*BAX*, *BCL2*), DNA repair (*DDB2*), and antioxidant defence (*SESN1*)^29^ (Supplementary Table 6b). Remarkably, in the subset of 21 CLs wild type for *TP53* and resistant to MI-773, we observed a lower expression level of these *TP53*-related genes (*MDM2*, *BCL2*, *BAX*, and *TP53*) compared with wild type *TP53* CLs sensitive to MI-773. The expression of these genes was similarly low in wild type and mutated *TP53* CLs resistant to MI-773, except for the *TP53* level that was high in the mutated group (Supplementary Fig. 2).

Next, we investigated the feasibility to predict response to MI-773 using molecular-based models. We tested whether unsupervised hierarchical clustering could result in the molecular classification of the 237 CLs into clusters with different response rates toward MI-773. For this, we used 237 out of the 316 differentially expressed genes with a consistent fold difference (>±0.5). The analysis showed three clusters of CLs with significant differences in MI-773 response rate (proportion test *p* = 2E^−04^) (Fig. 4c, Supplementary Table 7). Cluster 2 had the highest response rate (52%, 17/33), followed by cluster 1 (31%, 40/129) and cluster 3 (13%, 10/75). The mean MI-773 Abs IC_50_ of the sensitive CLs (<10 µM) in each of the three clusters is similar (2–3 µM), and that of the resistant CLs is a factor 6–8 higher (Supplementary Table 7). However, we observed that the clusters were largely driven by the histological origin of the CLs: solid vs. haematological tumour types, exemplified by cluster 2 consisting of only leukaemia and lymphoma CLs (Fig. 4c). On the other hand, the composition of cluster 1 is rather broad; basically, with all tumour types present in the CL panel. Interestingly, it also comprised all sensitive and resistant CLs from CNS, melanoma, renal and prostate cancer, sarcoma, SCLC, and mesothelioma. Cluster 3 contains the remainder of the CLs: liver, gastric, head and neck cancer, NSCLC, ovary, breast, uterus, oesophagus, and bladder cancer.

We, therefore, preferred to explore the feasibility of a comprehensive gene expression-based scoring system to predict response to MDM2 inhibition. For that, we selected the 11 genes related to the p53-signalling pathway that were the most significantly associated with MI-773 sensitivity. For each of the 237 CLs, we calculated a predictive score consisting of the average expression value of the 11 genes, and we set up a cut-off at 7.5 to predict sensitivity. We showed first that the predictive score was strongly associated with the *TP53* mutation status of the CLs (Supplementary Table 8): 53 out of 74 (72%) CLs with wild type *TP53* had a high score (>7.5) and 115 out of 163 CLs (70%) with *TP53* mutations had a low score (≤7.5) (Fisher’s exact test, *p*-value < 1.38E^−09^). The regression analysis showed that the predictive score was inversely correlated with the MI-733 Abs IC_50_ values across the 237-CL panel (Spearman *ϼ* = −0.6, *p*-value < 2.2E^−16^, upper plot of Fig. 4e). Notably, the predictive score also correlated with the MI-773 Abs IC_50_ values in the wild type *TP53* CL subset (Spearman *ϼ* = −0.7, *p*-value < 2.26E^−12^, lower plot of Fig. 4e). Overall, 128/136 CLs (94%) with a predictive score of ≤7.5 were resistant, whereas 59/101 CLs (58%) with a predictive score of >7.5 were sensitive (Fisher’s exact test *p*-value < 2.2E^−16^, Supplementary Table 9a). Several options and combinations were explored during the classifier development, particularly reduction of the number of genes, but the 11 genes together provided the most robust classifier.

Studying the performance of the predictive score in both wild type and mutated *TP53* CLs separately showed that the predictive score was particularly useful in wild type *TP53* CLs. Subdividing the 237 CLs into two groups, wild type *TP53*/high score and mutated *TP53*/low score, respectively, increased the accuracy of the prediction considerably for the wild type *TP53*/high score CLs from 58% to 91% and only slightly for the mutated *TP53*/low score CLs from 94% to 97% (Supplementary Table 9b, c).

The assumption that a decisional tree consisting of the combination of the *TP53* mutation status, and the predictive score would better predict sensitivity and resistance to MDM2 inhibition was tested with the MI-773 and Nutlin-3a sensitivity datasets (GDSC1 dataset). With this approach, 53 CLs were predicted sensitive to MI-773 (wild type *TP53* and a predictive score >7.5) and 184 CLs were predicted resistant (*n* = 163 with *TP53* mutated and 21 wild type *TP53* with a predictive score ≤7.5) (Fig. 4f, left). The sensitivity and specificity of the decisional tree test were 91% and 76%, respectively. It showed overall a superior positive predictive value (91%) to identify CLs sensitive to MI-773 than a negative one for the identification of resistant ones (76%) (Supplementary Table 10).

Molecular and drug sensitivity data of Nutlin-3a were available for 537 CLs from GDSC1. In this dataset, 98 CLs were also present in our 274-CL panel tested for MI-773 and were, therefore, not considered for the following validation study, leaving a final panel of 439 CLs. Like for MI-773, the predictive score for these CLs was strongly associated with *TP53* mutation status (two-sided Wilcoxon test *p*-value < 2.22E^−16^) (Supplementary Fig. 3a). We also validated that the predictive score negatively correlated with Nutlin-3a Abs IC_50_ in all CLs and in wild type *TP53* CLs only (Spearman *ϼ* = −0.5, *p*-value < 2.2E^−16^ and *ϼ* = −0.5, *p*-value = 4.5E^−9^, respectively (Supplementary Fig. 3b). Using the decisional tree, 109 out of 439 (25%) CLs were predicted to be sensitive to Nutlin-3a and 330/439 (75%) resistant (Fig. 4f, right). By taking a cut-off at the 30th percentile, 84 out of the 109 CLs (77%) were predicted sensitive to Nutlin-3a-Abs IC_50_ values ≤19.7 µM, whereas 283 out of 330 (86%) were predicted resistant with Nutlin-3a Abs IC_50_ > 19.7 µM. The decisional tree’s sensitivity and specificity for predicting Nutlin-3a sensitivity or resistance were 95% and 43%, whereas the positive predictive value for sensitivity was 77% and the negative one identifying resistance was 83% (Supplementary Table 11).

We finally tested the association between (a) the *TP53* mutation status, (b) the predictive score or (c) the combination of both for association with Abs IC_50_ for the other *TP53* related small molecules in the databases. As shown before, *TP53* mutation status was strongly associated with CL Abs IC_50_ of Nutlin-3a (GDSC2) and RG-7112 (Wilcoxon test *p*-value < 2.2E−16), to a lesser extent with YH239-EE, PRIMA-1MET, MIRA-1, JNJ-26854165 but not with NSC-207895 (Wilcoxon test *p*-value ≤ 0.05) (Supplementary Table 12). A similar sequence of compounds was found for the association between the predictive score and CL Abs IC_50_. However, we also noticed that the classification with the predictive score alone better predicts sensitivity to PRIMA-1MET, MIRA-1 and NSC-207895 compounds than the *TP53* mutation status. The association was weaker for YH239-EE (Wilcoxon test *p*-value = 0.02) and absent for JNJ-26854165. In wild type *TP53* CLs only, the association was well preserved for Nutlin-3a and RG-7112, borderline significant for PRIMA-1MET, MIRA-1, NSC-207895 and not significant for YE239-EE and JNJ-26854165. Thus, the decisional tree combining *TP53* mutation status and the predictive score showed a significant association with all compounds (Wilcoxon test *p* < 0.05), which was much more pronounced for compounds targeting the p53–MDM2 interaction than for the others.

## Discussion

Various classes and generations of small-molecule MDM2 inhibitors have been developed over the last 20 years, of which Nutlin-3a was the first potent inhibitor^9,10^. The current knowledge of the anti-tumour activity spectrum of these compounds is not exhaustive. Although tumours with gene amplification and overexpressing *MDM2* and *MDM4*, the negative regulators of p53^30,31^, are obvious candidates for such therapy, other tumours should be targetable with this type of compound.

Testing MI-773 in a wide range of CLs demonstrated that MI-773 has potential for a broad range of solid tumour types and haematological malignancies. Melanoma, renal cancer, sarcoma, gastric cancer, leukaemia, and lymphoma were the most sensitive cancer types. In line with the previously reported mechanism of actions^32,33^, our COMPARE correlation analyses, a large-scale exercise using hundreds of drug sensitivity datasets annotated for the mechanisms of action, showed that the anti-tumour profile of MI-773 was similar to those of Nutlin-3a and RG-7112, other antagonists targeting the p53–MDM2 interaction. In contrast, the study showed that the MI-773 inhibitory profile was poorly correlated with those of compounds targeting the p53 pathway via another mechanism of action (MIRA-1, PRIMA-1MET, JNJ-26854165 or NSC-207895). The comparison study also supported that MI-773 had a superior potency over Nutlin-3a.

With an extended pharmacogenomic analysis, we validated that *TP53* mutations can be considered, irrespective of their type or localisation, as a universal marker of resistance to inhibitors targeting p53–MDM2 interactions. This was not the case for the other p53-related compounds for which the *TP53* mutation status was only poorly predictive. Our study also demonstrated that the sensitivity of wild type *TP53* CLs to MI-773 varied considerably; some CLs were strongly resistant. The recently reported results of the inhibitor of p53–MDM2 DS-3032b are in line with our data. *TP53* mutations predicted overall resistance. Only a few *TP53*-mutated CLs were sensitive to MDM2 inhibition, and just like our results, a part of the wild type *TP53* CLs was strongly resistant to the compound^34^. These observations are in accordance with the work of Donehower et al. ^5^, demonstrating that more than 90% of *TP53*-mutant cancers exhibit second allele loss of expression, either by mutation, chromosomal deletion, or copy-neutral loss of heterozygosity, leading to alteration of the p53 protein level in most cases. Thus, the type of *TP53* mutation and the level of p53 determine the sensitivity of *TP53*-mutated CLs for p53–MDM2 inhibitors. In wild type *TP53* CLs, the large variety in drug response implies that additional factors determine the sensitivity to MDM2 inhibition.

The large size and the transcriptome annotation of our CL panel allowed us to successfully run a differential gene expression analysis to identify additional predictors of MI-773 sensitivity. To this end, we did not select wild type *TP53* CLs only since the number (*n* = 74) was an explicit limitation to obtain significant results with adjusted *p*-values. By considering all CLs, we identified hundreds of differentially expressed genes between groups of CLs that were sensitive or resistant to MI-773. Our work supports that unsupervised hierarchical clustering using these genes was not satisfying to classify CLs since tumour type rather than p53–MDM2 functionality was the basis of the clustering. It had hardly predictive value because each cluster comprised sensitive and resistant CLs. The sensitive CLs were mainly wild type *TP53*, and >80% of the resistant CLs had *TP53* mutations.

Pathway analysis of differentially expressed genes highlighted that the top predictors of MI-773 response were 11 upregulated genes closely related to the p53 signalling pathway, including *MDM2* itself. Four genes (*CDKN1A*, *DDB2*, *MDM2*, *CCNG1*) were retrieved in the 20 most significantly upregulated genes in wild type *TP53* tumours in the TCGA database^5^. Another four genes (*BAX*, *MDM2*, *ZMAT3*, and *CDKN1A*) belonged to the 10 upregulated genes in circulating wild type *TP53* leukaemia cells of patients treated with the MDM2-antagonist RG7112 and were absent in mutated *TP53* leukaemia cells^35^.

To predict MI-773 response, we decided to test a supervised approach to develop a comprehensive gene expression-based scoring system named “predictive score” based on these 11 genes. The low number of genes and their positive association with the sensitivity to the compound present advantages for better applicability in the clinic. Combining the predictive score and the *TP53* status strengthened the identification of CLs sensitive for MI-773 and other p53–MDM2 inhibitors Nutlin-3a and RG-7112. Our results further suggest that the predictive score alone was also applicable to mutant p53-reactivating small molecules (MIRA-1 and PRIMA-1MET) and the MDMx inhibitor NSC-207895, but not to MDM2–proteasome interaction inhibitor JNJ-26854165 (Serdemetan). The predictive value of the score has now to be validated in patient tumour samples and in a prospective study. Most probably, some adjustments will be necessary for assessment in tumours containing stroma. Similarly, Zhong et al. ^36^ developed a 4-gene mRNA MDM2-antagonist therapy predictive signature, which is under investigation, and Ishizawa et al. ^34^ developed a 175-gene signature. The 175 genes represented the top 1% of genes upregulated in MDM2-inhibitor sensitive CLs. The Spearman correlation between in vitro sensitivity and the 175-gene signature score obtained by Ishizawa et al. ^34^ was similar to ours (*ϼ* = 0.67). An additional 1500-gene signature focusing on AML did not improve the prediction, suggesting that more is not better and is not feasible in a clinical setting. In general, biomarkers are not always used in current clinical studies with MDM2 inhibitors, a part used *TP53* for patient selection^9^.

Apart from the results of our study, other findings should be considered in the development of MI-773. Kim et al. ^37^ showed the efficacy of MI-773 in glioblastoma. However, further experiments with orthotopically implanted PDX models demonstrated that the low penetration through the blood–brain barrier limited MI-773’s efficacy. The compound is a substrate of P-glycoprotein limiting its distribution to the brain by active efflux and the authors proposed a combination therapy with efflux transporter inhibitors^38^. In another aspect, it was observed thatw the treatment of dedifferentiated liposarcoma patients with MI-773 can cause the emergence of resistant *TP53*-mutated clones^39^. For long-term control of the disease, combination therapies have been proposed. The combination of MI-773 and pimasertib, an oral MEK1/2 inhibitor, was promising in wild type *TP53* preclinical melanoma models in vivo^21^. The subsequent Phase I clinical study showed that the safety profile of the combination was consistent with the safety profiles of both drugs individually, and preliminary anti-tumour activity was observed^21^. Other combinations of MDM2 inhibitors with chemotherapeutic or targeted agents are being explored in early clinical studies such as cytarabine, BCL-2 (venetoclax), BRAF (trametinib), MEK (dabrafenib), CDK4/6 (palbociclib) inhibitors, and immune checkpoint inhibitors^23,40,41^.

The pharmacogenomic work described here was performed in the context of the development of our “Cancer Data Mining” platform. Our study demonstrated that the drug and molecular information available on our platform is relevant for validating the mechanism of action of compounds and targets, discovering candidate tumours for therapy, and early identification of predictive biomarkers. The concept of our approach is based on testing associations between the sensitivity of CLs for a drug and the basal gene expression levels or genomic alteration profile of the CLs, which recently was successful in revealing mechanisms of action of drugs and protein targets^42^. This approach requires large sets of molecularly annotated preclinical models. It is crucial to first understand the mechanism behind the sensitivity and resistance of new anti-cancer agents before testing the compound in vivo in tumour models, e.g., PDX. Given the inter- and intra-tumour heterogeneity, in vitro screening of compounds should preferably be performed in hundreds of CLs, allowing statistical analysis. The accuracy and reliability of OMICS data, usually obtained from accredited laboratories, is also a prerequisite for the success of such studies. The microenvironment of tumour models in vivo will add another layer of complexity to understand the effect of a drug on sensitivity and resistance.

In conclusion, the combination of in vitro preclinical investigations and pharmacogenomic studies extended the therapeutic options of the p53–MDM2 inhibitor MI-773 to a broad range of wild type *TP53* haematological and solid tumour types. *TP53* status combined with the gene expression-based predictive score may help to infer tumour response to this inhibitor. Additional studies in vivo are needed to validate the anti-tumour effect and assess MI-773 toxicity and other studies in independent cohorts to validate the predictive score. Furthermore, combination studies with p53–MDM2 inhibitors are inevitable and warrant further investigation.

## Methods

### Drugs

MI-773 (SAR405838, MI-77301) (Catalogue No. S7649) and Nutlin-3a (Catalogue No. S8059) were purchased from Selleckchem (Munich, Germany). The drugs were dissolved in DMSO to obtain a stock solution, which was kept at 4 °C, and immediately before use further diluted with culture medium to concentrations required for the in vitro experiments.

### Human tumour CLs

Two hundred and seventy-four CLs from the Oncotest GmbH repository (Freiburg Germany, since 2015 Charles River Discovery Research Services (DRS) Germany GmbH) were used in the study (Supplementary Table 1). Cells were grown at 37 °C in a humidified atmosphere with 5% CO_2_ in the medium recommended by the provider (Supplementary Table 1). The annotations of the CLs were performed via the Charles River DRS database and the COSMIC cancer cell line database^43^. Tumour cell line authenticity was confirmed by short tandem repeat analysis at DSMZ (Braunschweig, Germany). The CLs were mycoplasma free.

The 274-CL panel consisted of 222 CLs derived from solid cancers including 68 CLs (25%) from the digestive system (16 CRC, 16 stomach, 15 pancreases, 11 liver hepatomas, five liver cholangiocarcinomas, and five oesophagi), 49 (18%) from the urogenital tract (14 kidneys, 11 ovaries, eight bladders, five prostate, and 11 uteri), 43 (15%) from the respiratory system (28 non-small cell lung (NSCLC), 10 small cell lung (SCLC) and five mesotheliomas), 15 (5%) from breast cancer, 13 (5%) from sarcomas (one Ewing, three osteosarcomas, nine soft tissue), 12 (4%) from head and neck (HNSC), 12 (4%) from melanomas, and nine (3%) from the central nervous system (CNS, glioblastomas) and one epidermoid carcinoma CL (A431) classified as skin tumour (from vulva). The 52 haematological CLs included 27 leukaemia (10%) (11 AML, eight acute lymphocytic leukaemia (ALL), seven chronic myeloid leukaemia (CML), one chronic lymphocytic leukaemia (CLL)) and 25 lymphomas (9%) (11 multiple myelomas (MM), four Burkitt, five diffuse large B-cell (DLBC), one Hodgkin and four unclassified).

### Cell proliferation assay

A modified propidium iodide assay was used to screen the CLs, growing in a 2D-monolayer culture, for sensitivity to MI-773^44^. Briefly, cells were harvested from exponential phase cultures, counted, and seeded in 96-well flat-bottom microtiter plates at a cell density of 4000–30,000 cells/well, dependent on the growth rate of the CLs. Haematologic and CLs from SCLC were grown in suspension cultures. After a 24-h recovery period to allow the cells to resume exponential growth, 10 μl of culture medium (six control wells/cell line/plate) or culture medium with MI-773 was added. The compound was serially diluted and applied at ten concentrations in half-log increments from 0.001 up to 30 µM in singlicate and treatment continued for four days. Cells were then washed with 200 µl phosphate-buffered saline to remove dead cells and debris. Next, 200 µl of a solution containing 7 µg/ml propidium iodide and 0.1% (v/v) Triton X-100 was added to the wells. After 1–2 h incubation at room temperature, fluorescence was measured using an Enspire^®^ multimode plate reader (excitation *λ* = 530 nm, emission *λ* = 620 nm; Perkin Elmer, Rodgau, Germany) to quantify the number of viable cells.

Drug effects on cell proliferation and survival were expressed as Test/Control × 100 (%) values, using the mean fluorescence signal of treated and control wells. IC_50_ values, absolute and relative, were calculated by a four-parameter non-linear curve fit (Charles River Data Warehouse Software).

The median of all individual IC_50_ values determined the overall potency of MI-773. If an individual IC_50_ value could not be determined within the examined dose range (because the compound was either too active or lacked activity), the lowest or highest concentration studied was used for the value calculation.

### Molecular databases

Most of the CLs tested for MI-773 were characterised by 4HF Biotec GmbH as described below. CLs were characterised by next-generation sequencing for whole-exome mutations, single nucleotide polymorphism (SNP) array (Affymetrix Genome-Wide Human SNP Array 6.0 (SNP6.0)) for SCNA and gene expression array for transcriptomic profiles (Affymetrix Human Genome U133 Plus 2.0 Array). Missing profiles and additional data used for the biomarker validation were obtained from the Cancer Cell Line Encyclopedia^45^ (downloaded 2017). In this case, processed whole-exome mutation data were directly downloaded. CEL files of both SNP6.0 and Human Genome U133 Plus 2.0 Array were processed together with the internal CL profiles to limit batch effects.

### DNA and RNA isolation and purification

DNA and RNA were extracted, according to the adapted protocols previously described^46^. In brief, cells were placed on ice, washed three times with cold phosphate-buffered saline to remove any traces of medium. Adherent cells were removed from the cell culture dish using sterile cell scrapers. Cell pellets were frozen in liquid nitrogen immediately after the last washing step and stored at −80 °C until extraction. On the day of DNA and RNA extraction, the frozen cell pellets were placed on ice, and then the appropriate lysis buffer was added.

For DNA extraction, proteinase K buffer (Qiagen, Hilden, Germany) was added to the lysed cell suspension, and the mixture was incubated overnight at 55 °C. The lysates were digested with DNase-free RNase (Qiagen, Hilden, Germany), the DNA was extracted with phenol/chloroform/isoamyl alcohol and precipitated with ethanol. The pellets were washed and resuspended in TE buffer (Tris 10 mM pH8, EDTA 0.1 mM pH8). The DNA integrity of each preparation was checked on a 1.3% agarose gel, and the quantity and purity were analysed with a NanoDrop 2000 spectrophotometer (Thermo Fisher Scientific, RRID:SCR_008452).

RNA was extracted using the mirVana^™^ miRNA isolation kit (Ambion, Carlsbad, CA, USA) according to the manufacturer’s instructions. The RNA quality and purity were controlled with a NanoDrop 2000 spectrophotometer (RRID:SCR_018042), and RNA integrity by a Bioanalyzer (Agilent Technologies, RRID:SCR_018043).

### Gene expression

Microarray gene expression profiles were generated by using the Affymetrix Human Genome U133 Plus 2.0 GeneChip arrays according to Affymetrix recommendations at the AROS laboratory (now Eurofins Genomics Europe Genotyping A/S, Denmark). CEL files were subjected to internal quality control measures using Affymetrix RLE/NUSE. Gene expression signal values were extracted directly from the CEL files using the GeneChip robust multi-array average (gcrma) expression algorithm. This was achieved using the R package “gcrma” from the Bioconductor project^47^. Signal values were log_2_ transformed.

### Exome mutations

Whole-exome sequencing was performed at the GATC laboratory (now Eurofins Genomics, Konstanz, Germany). The enriched exonic DNA was sequenced on an Illumina Hiseq2000, 2500 or 4000 (paired-end reads, RRID:SCR_016383, SCR_016386) with a minimum coverage of 100×.

Raw reads were subjected to FastQC (RRID:SCR_014583) to calculate read quality metrics^48^. After the alignment to the human reference genome (Burrows–Wheeler Aligner version 0.7.17)^49^, the quality of BAM files was assessed by Qualimap 2.2.1 (RRID:SCR_001209) to obtain the percentage of mapped reads and coverage of reads to the targeted exons (as defined by Agilent)^50^. The mapped reads were recalibrated with GATK (RRID:SCR_001876) *BaseRecalibrator* function after duplicates removal and indel local realignment^51^. Reads mapped around indels were realigned used the GATK’s *IndelRealigner* function before performing the variant calling step. Variants were detected independently using three different variant callers: the GATK *UnifiedGenotyper*, the combination of Samtools (RRID:SCR_002105) *mpileup* and *bcftools*, and Freebayes (RRID:SCR_010761)^52–54^. Only variants identified by all three tools, showing a minimum number of variant-supporting reads of three and a minimum variant frequency of 5%, were further analysed. Candidate mutations were annotated with SnpEff (RRID:SCR_005191) by selecting only single-nucleotide variants and insertions/deletions (Indels) with a high or moderate protein impact from UCSC or Ensembl transcripts^55^, and by filtering out known polymorphisms from annotation databases if a variant (1) has at least three allele counts from Hapmap (RRID:SCR_002846) or CGI 69 genomes or EVS + 1000 Genomes or (2) shows more than 5% of a minor allele in at least one population from dbSNP (RRID:SCR_002338). The quality control of variant detection analysis was evaluated with SnpEff by computing and validating the transition/transversion ratio from SNP found in exons.

### Somatic copy number alterations

The detection of chromosomal alterations was performed with the Affymetrix SNP6.0 array following the standard protocol recommended by the manufacturer at the AROS laboratory (now Eurofins Genomics Europe Genotyping A/S, Denmark). According to Affymetrix guidelines, CEL files were subjected to internal quality control measures, including contrast quality control and MAPD threshold using Genotyping Console Software (Thermo Fisher Scientific, RRID:SCR_008452). SCNA values (0–14) were determined from the CEL files using the PICNIC algorithm^56^.

### Statistical analysis methods

Statistical analyses were carried out using The R Project for Statistical Computing (R statistical environment version 3.4.4, RRID:SCR_001905) and associated packages from Bioconductor in the Linux operating system (Ubuntu, 16.04.4)^57^. *p*-values < 0.05 were considered statistically significant. Data are represented as means ± standard deviation (SD), and median (interquartile range (IQR)). For biomarker screening, drug response data were treated either as continuous variables using absolute IC_50_ (Abs IC_50_) or as categorical variables (with two groups of highly sensitive plus intermediate sensitive versus resistant CLs, at the Abs IC_50_ 30th percentile (cut-off = 10.7 µM)). To identify whole-exome mutations and SCNA associated with drug response, for each gene, samples with at least one alteration (whole-exome mutation or SCNA ≥ 8 or SCNA = 0) were given the value 1 and no alteration was given 0, resulting in matrices of binary representation. Next, binary matrices of genomic alterations (exome mutations, copy number variations) were used for a gene per gene statistical analysis for association with drug sensitivity (Abs IC_50_ and drug sensitivity groups). Wilcoxon rank-sum test was performed between continuous variables (Abs IC_50_) and binary categorical variables (mutated versus not mutated or high SCNA versus no SCNA). Fisher’s exact test was used for independence between two categorical variables. For any test between two continuous variables, statistical dependence between the rankings of the two variables was evaluated by Spearman’s rank correlation test.

### Differential gene expression analysis

The association between gene expression and drug Abs IC_50_ or sensitivity groups was tested using Limma (package version 3.34.9)^58,59^, *t*-test and Spearman tests. A cut-off was set at 10.7 µM to dichotomise sensitive CLs to MI-773 from resistant ones (which corresponds to 30% of the 237-CL panel). The gene expression profiles were curated by removing probe sets with lack of expression (probe sets < 5 were excluded), resulting in 31,751 probe sets with consistent expression. They were tested for an association between their expression levels and MI-773 Abs IC_50_ values. Probe sets significantly associated with sensitivity to MI-773 in all three statistical tests (adjusted *p* < 0.05) were then filtered by Jetset curation^52^ to retain unique gene transcripts. Those with a pronounced differential expression between CLs sensitive and resistant to MI773 (log_2_ fold difference > ±0.5) were used to perform unsupervised hierarchical clustering using the function *clustering* from the EMA library (version 1.4.4). The ComplexHeatmap package (version 2.4.3) was used to create and visualise the clusters and heatmaps.

For each statistical test with multiple testing hypotheses, *p*-values were adjusted with Benjamini and Hochberg correction as a conservative method for probability thresholding to control the occurrence of false positives^60^. Lollipop mutation diagrams were generated using the lollipops software^61^. To gain insight into the nature of the gene subset that were positively associated with MI-773 sensitivity, Kyoto Encyclopedia of Genes and Genomes (KEGG) and the GO BP-enrichment pathway analysis was carried out with *enrichKEGG* and *enrichGO* (*ont* = *“BP”*), respectively in clusterProfiler (RRID:SCR_016884, version 3.6.0)^62^.

### Cancer data miner platform

Anti-tumour activity and genomic data were unified in an in silico platform called “4HF Cancer Data Miner”, dedicated to accelerate R&D efforts in drug discovery and development, optimise target evaluation, identify promising clinical therapeutic areas, and discover putative predictive biomarkers.

### Reporting summary

Further information on research design is available in the Nature Research Reporting Summary linked to this article.

## Supplementary information

Supplementary Information

Reporting Summary

## Data Availability

For the biomarker analysis of MI-773: Affymetrix Human Genome U133 Plus 2.0 transcriptomic data have been deposited in Gene Expression Omnibus (GEO) under the accession code GSE152529. The Affymetrix SNP6.0 data used have been deposited in GEO under the accession code GSE178763 or were obtained from CCLE (https://depmap.org/portal/ccle). The whole exome sequencing data been deposited are accessible under BioProject accession PRJNA750602 or were obtained from CCLE (https://depmap.org/portal/ccle). For the validation of the scoring system using Nutlin-3a compound, all molecular data were obtained from CCLE (https://depmap.org/portal/ccle).

## Code availability

The code used to analyse the genomic alterations is available upon request from the corresponding author.
