# Supplementary material for: Pharmacogenomics characterization of the MDM2 inhibitor MI-773 reveals candidate tumours and predictive biomarkers
Source: NPJ Precis Oncol. 2021 Oct 28;5:96. doi: 10.1038/s41698-021-00235-7 (PMC8553758; doi:10.1038/s41698-021-00235-7)
Supplement: Supplementary file 1 — Supplementary Information [file 41698_2021_235_MOESM1_ESM.pdf]

Supplementary Appendix

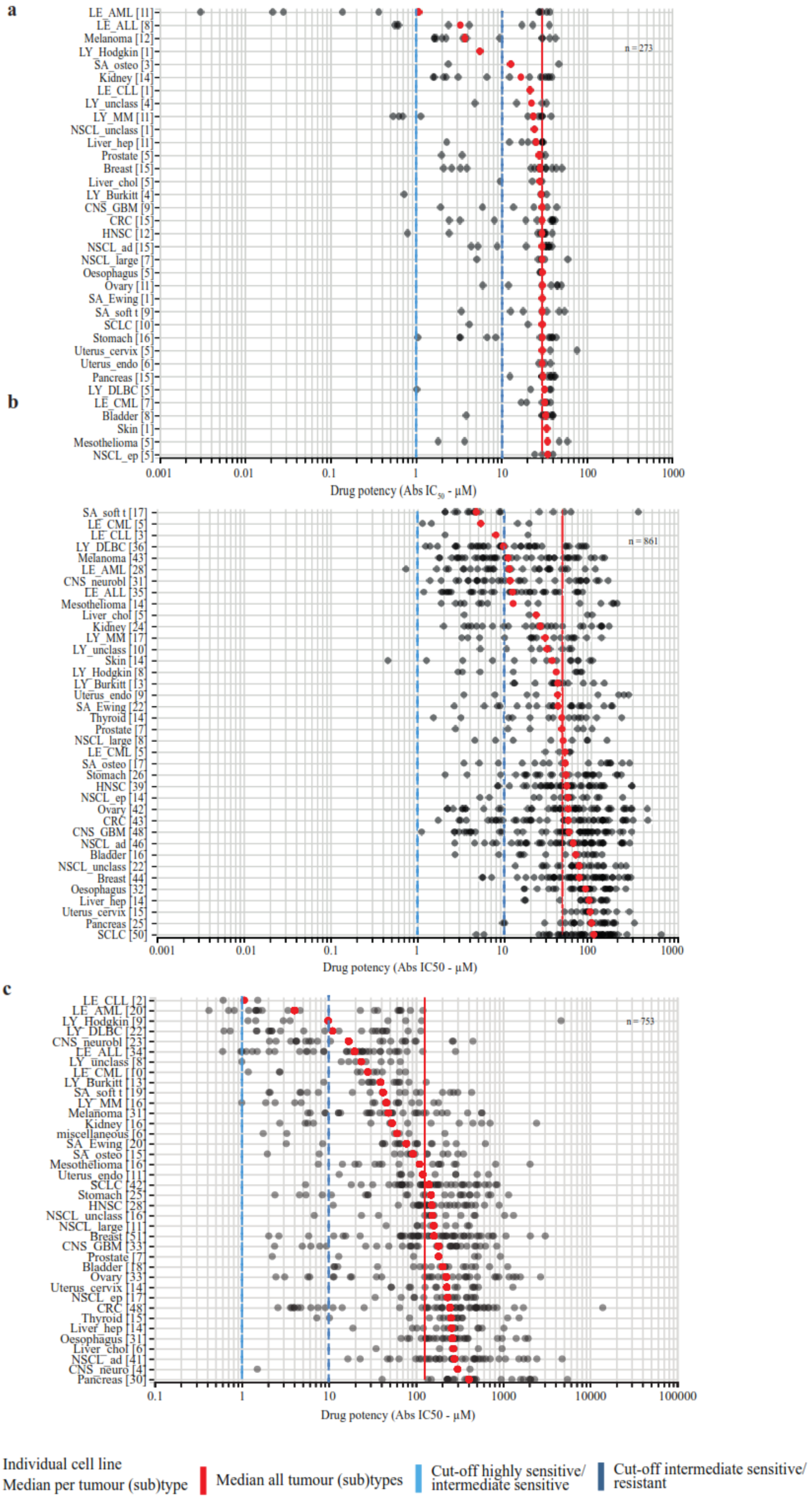

Supplementary Figure 1. Scatter plot of Nutlin-3a Abs IC_{50} value for each CL across cancer (sub)types (x-axis).

a. 4HF Biotec internal dataset **b-c.** GDSC1 and GDSC2 datasets. X-axis: Nutlin-3a Abs IC_{50} value per CL, y-axis: the histological (sub)types sorted from top to bottom by increasing median Abs IC_{50} values. The red dots are the median Abs IC_{50} value for each tumour (sub)type, and the red line is the overall median Abs IC_{50} value. The blue lines are the cut-off values of 1 and 10 µM. Between brackets the total number of CLs within a tumour (sub)type. Abbreviations: CNS_GBM central nervous system: glioblastoma, CRC colorectal cancer, HNSC head & neck squamous cell, LE_ALL acute lymphoblastic leukaemia, LE_AML acute myeloid leukaemia, LE_CLL chronic lymphocytic leukaemia, LE_CML chronic myelogenous leukaemia, Liver_chol Liver_cholangioma, Liver_hep liver_hepatocellular, LY_Burkitt lymphoma_Burkitt, LY_DLBC lymphoma_Diffuse large B cells, LY_Hodgkin lymphoma_Hodgkin, LY_MM lymphoma_multiple myeloma, LY_unclass lymphoma_unclassified, NSCL_ad non-small cell lung_adenocarcinoma, NSCL_ep non-small cell lung_epidermoid, NSCL_large non-small cell lung_large cells, NSCL_unclass non-small cell lung_unclassified, SA_Ewing Sarcoma Ewing, SA_osteo osteosarcoma, SA_soft t sarcoma soft tissue, SCLC small cell lung, uterus_endo uterus_endometrium.

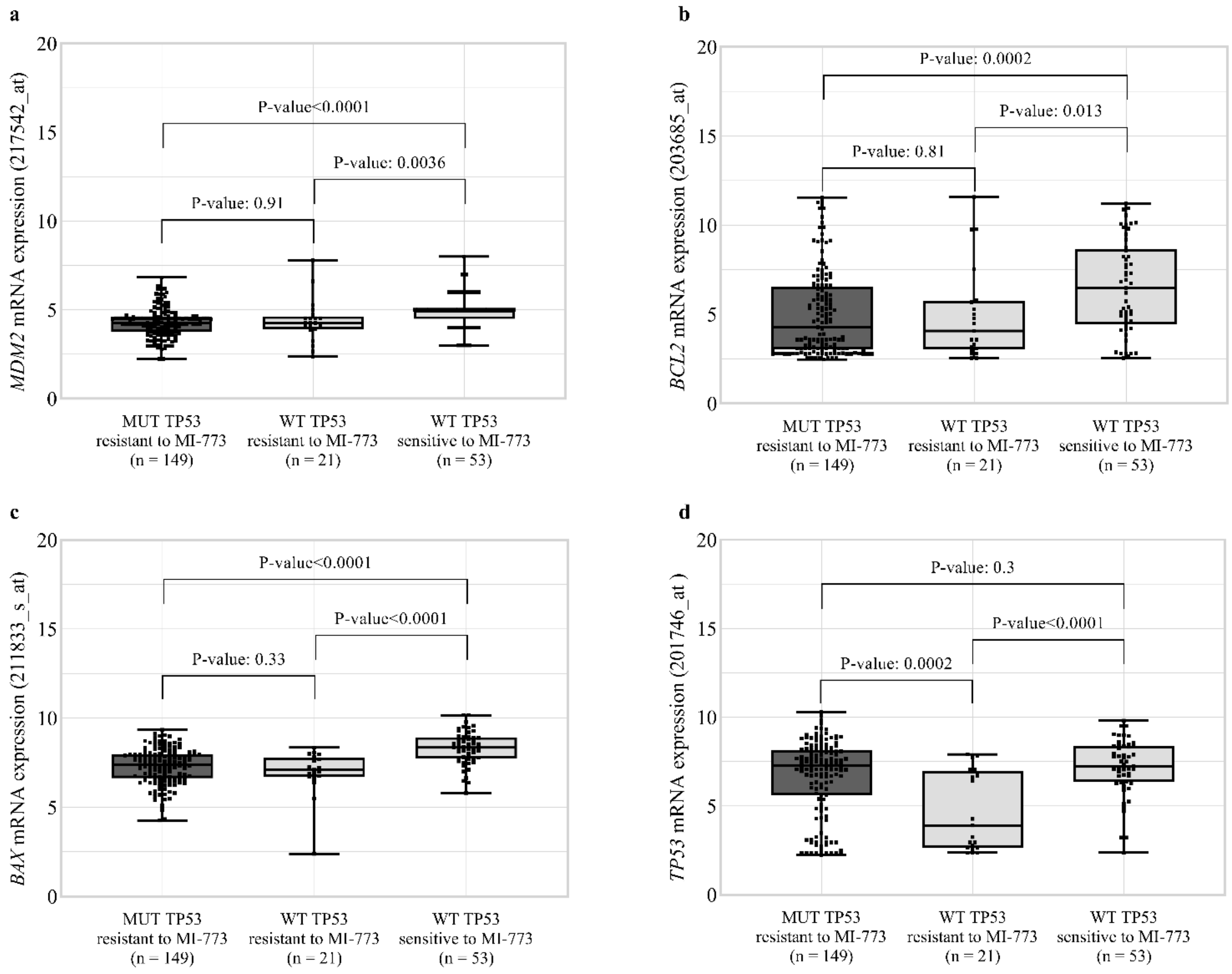

Supplementary Figure 2: mRNA expression of *MDM2, BCL2, BAX,* and *TP53* stratified by *TP53* mutation status and the response to MI-773.

Boxplots showing mRNA expression of **a.** *MDM2*, **b.** *BCL2*, **c.** *BAX*, **d.** *TP53* (Data: Affymetrix HGU133 Plus2.0 microarray, probe sets selected using Jetset best, see Methods section) in mutated *TP53* (MUT *TP53*) CLs and wild type *TP53* (WT *TP53*) CLs, both resistant to MI-773, and compared with wild type *TP53* (WT *TP53*) CLs sensitive to MI-773. P-values were obtained from the Wilcoxon test. Box plot: minimum, 25th percentile, median, 75th percentile, and maximum, data points are plotted as black dots.

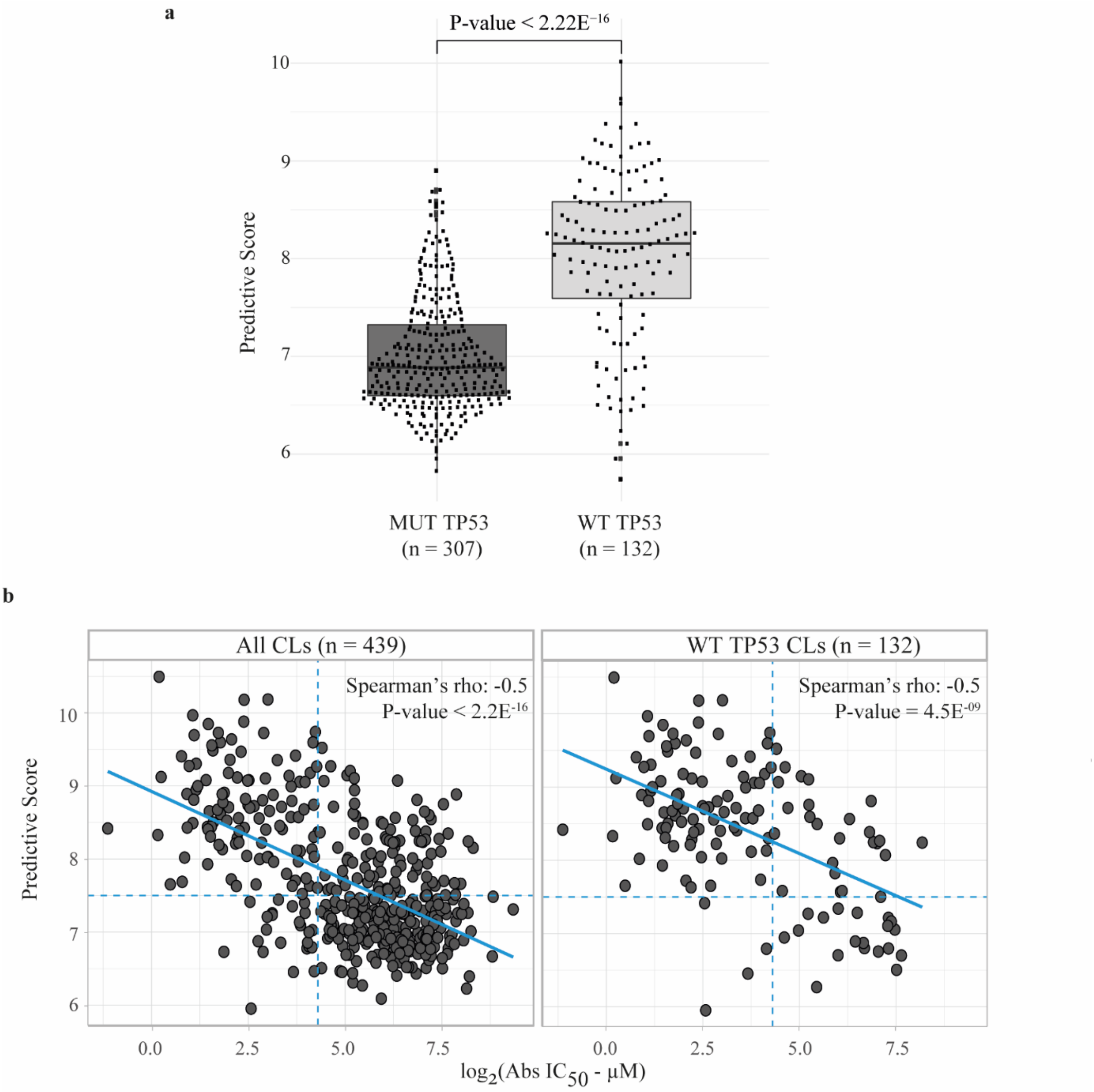

Supplementary Figure 3. Testing predictive score in theNutlin-3a dataset (GDSC1).

a. Boxplot of predictive scores in wild type *TP53* (WT *TP53*) and mutated (MUT *TP53*) in the 439 CLs treated with Nutlin-3a used for predictive score validation. P-value was obtained from the Wilcoxon test. Box plot: minimum, 25th percentile, median, 75th percentile, and maximum, data points are plotted as black dots.

b. Spearman correlation analysis between Nutlin-3a Abs IC_{50} values and the gene expression-based predictive score. Left: all 439 CLs, right: wild type *TP53* CLs only (n=132).

Supplementary Table 1: Characteristics of the 274-CL panel, molecular data availability, culture conditions, resources designation and MI-773 Abs IC_{50}.

Tumour type	Tumour subtypes (annotation)	Cell lines	Molecular data availability**	Culture medium	Resource	Catalogue number	MI-773 (Abs IC_{50}, µM)
Bladder	Bladder	BXF_1036	Yes	RPMI 1640; 10% FCS; 0.05 mg/ml Gentamycin	CRL FR*		19.716
Bladder	Bladder	BXF_1228	Yes	RPMI 1640; 10% FCS; 0.05 mg/ml Gentamycin	CRL FR*		12.027
Bladder	Bladder	BXF_1352	Yes	RPMI 1640; 10% FCS; 0.05 mg/ml Gentamycin	CRL FR*		10.288
Bladder	Bladder	BXF_5637	No	RPMI 1640; 10% FCS; 0.05 mg/ml Gentamycin	DSMZ	ACC 35	30.821
Bladder	Bladder	KU-19-19	Yes	RPMI 1640; 10% FCS; 0.05 mg/ml Gentamycin	DSMZ	ACC 395	5.144
Bladder	Bladder	RT112	Yes	RPMI 1640; 10% FCS; 0.05 mg/ml Gentamycin	DSMZ	ACC 418	1.491
Bladder	Bladder	SW1710	Yes	RPMI 1640; 10% FCS; 0.05 mg/ml Gentamycin	DSMZ	ACC 426	17.58
Bladder	Bladder	T24	Yes	RPMI 1640; 10% FCS; 0.05 mg/ml Gentamycin	ATCC	HTB-4	18.855
Breast	Breast	BT-474	Yes	RPMI 1640; 10% FCS; 0.05 mg/ml Gentamycin	DSMZ	ACC 64	27.541
Breast	Breast	BT-549	Yes	RPMI 1640; 10% FCS; 0.05 mg/ml Gentamycin	CLS	300132	22.707
Breast	Breast	CAL-51	Yes	DMEM; 10% FCS; 0.05 mg/ml Gentamycin	DSMZ	ACC 302	1.023
Breast	Breast	DU-4475	Yes	RPMI 1640; 10% FCS; 0.05 mg/ml Gentamycin	NCI		1.341
Breast	Breast	EVSA-T	Yes	RPMI 1640; 10% FCS; 0.05 mg/ml Gentamycin	DSMZ	ACC 433	14.317
Breast	Breast	HCC1937	Yes	RPMI 1640; 10% FCS; 0.05 mg/ml Gentamycin	DSMZ	ACC 513	15.17
Breast	Breast	JIMT-1	Yes	RPMI 1640; 10% FCS; 0.05 mg/ml Gentamycin	DSMZ	ACC 589	16.154
Breast	Breast	MAXF_401	Yes	RPMI 1640; 10% FCS; 0.05 mg/ml Gentamycin	CRL FR*		10.789
Breast	Breast	MCF 10A	No	DMEM/F12 5%Horse Serum; 0.5 mg/mL Hydrocortisone; 10mg/mL Insulin; 20ng/mL hEGF; 0.05 mg/ml Gentamycin	ATCC	CRL 10317	0.518
Breast	Breast	MCF7	Yes	RPMI 1640; 10% FCS; 0.05 mg/ml Gentamycin	NCI		0.425
Breast	Breast	MDA-MB-231	Yes	RPMI 1640; 10% FCS; 0.05 mg/ml Gentamycin	ECACC	92020424	13.358
Breast	Breast	MDA-MB-453	Yes	RPMI 1640; 10% FCS; 0.05 mg/ml Gentamycin	ATCC	HTB-131	12.014

Breast	Breast	MDA-MB-468	Yes	RPMI 1640; 10% FCS; 0.05 mg/ml Gentamycin	ATCC	HTB-132	18.661
Breast	Breast	SK-BR-3	Yes	RPMI 1640; 10% FCS; 0.05 mg/ml Gentamycin	DSMZ	ACC 736	19.392
Breast	Breast	T47D	Yes	RPMI 1640; 10% FCS; 0.05 mg/ml Gentamycin	ECACC	85102201	13.371
Central Nervous System	Glioblastoma (CNS_GBM)	A172	Yes	RPMI 1640; 10% FCS; 0.05 mg/ml Gentamycin	ATCC	CRL-1620	0.356
Central Nervous System	Glioblastoma (CNS_GBM)	U-251MG	Yes	RPMI 1640; 10% FCS; 0.05 mg/ml Gentamycin	NCI		18.085
Central Nervous System	Glioblastoma (CNS_GBM)	CNXF_498	Yes	RPMI 1640; 10% FCS; 0.05 mg/ml Gentamycin	CRL FR*		1.037
Central Nervous System	Glioblastoma (CNS_GBM)	LN-229	Yes	RPMI 1640; 10% FCS; 0.05 mg/ml Gentamycin	ATCC	CRL-2611	7.601
Central Nervous System	Glioblastoma (CNS_GBM)	M059K	Yes	RPMI 1640; 10% FCS; 0.05 mg/ml Gentamycin	ATCC	CRL-2365	10.043
Central Nervous System	Glioblastoma (CNS_GBM)	SF268	Yes	RPMI 1640; 10% FCS; 0.05 mg/ml Gentamycin	NCI		16.417
Central Nervous System	Glioblastoma (CNS_GBM)	SF295	Yes	RPMI 1640; 10% FCS; 0.05 mg/ml Gentamycin	NCI		25.243
Central Nervous System	Glioblastoma (CNS_GBM)	SNB-19	Yes	RPMI 1640; 10% FCS; 0.05 mg/ml Gentamycin	NCI		17.347
Central Nervous System	Glioblastoma (CNS_GBM)	SNB-75	Yes	RPMI 1640; 10% FCS; 0.05 mg/ml Gentamycin	NCI		17.629
Colorectal Cancer	CRC	Caco-2	No	RPMI 1640; 10% FCS; 0.05 mg/ml Gentamycin	DSMZ	ACC 169	25.023
Colorectal Cancer	CRC	COLO-205	Yes	RPMI 1640; 10% FCS; 0.05 mg/ml Gentamycin	NCI		12.96
Colorectal Cancer	CRC	COLO-320	Yes	RPMI 1640; 10% FCS; 0.05 mg/ml Gentamycin	DSMZ	ACC 144	11.735
Colorectal Cancer	CRC	CXF_1103	Yes	RPMI 1640; 10% FCS; 0.05 mg/ml Gentamycin	CRL FR*		19.283
Colorectal Cancer	CRC	CXF_260	Yes	RPMI 1640; 10% FCS; 0.05 mg/ml Gentamycin	CRL FR*		13.112
Colorectal Cancer	CRC	CXF_280	Yes	RPMI 1640; 10% FCS; 0.05 mg/ml Gentamycin	CRL FR*		7.828
Colorectal Cancer	CRC	CXF_94	Yes	RPMI 1640; 10% FCS; 0.05 mg/ml Gentamycin	CRL FR*		13.531
Colorectal Cancer	CRC	DIFI	Yes	RPMI 1640; 10% FCS; 0.05 mg/ml Gentamycin	unknown		15.095
Colorectal Cancer	CRC	HCC2998	Yes	RPMI 1640; 10% FCS; 0.05 mg/ml Gentamycin	NCI		15.543
Colorectal Cancer	CRC	HCT116	Yes	RPMI 1640; 10% FCS; 0.05 mg/ml Gentamycin	NCI		0.645
Colorectal Cancer	CRC	HCT-15	Yes	RPMI 1640; 10% FCS; 0.05 mg/ml Gentamycin	NCI		13.696
Colorectal Cancer	CRC	HT-29	Yes	RPMI 1640; 10% FCS; 0.05 mg/ml Gentamycin	NCI		17.818

Colorectal Cancer	CRC	KM12	Yes	RPMI 1640; 10% FCS; 0.05 mg/ml Gentamycin	NCI		17.508
Colorectal Cancer	CRC	LOVO	Yes	RPMI 1640; 10% FCS; 0.05 mg/ml Gentamycin	ATCC	CCL-229	3.352
Colorectal Cancer	CRC	RKO	Yes	RPMI 1640; 10% FCS; 0.05 mg/ml Gentamycin	ATCC	CRL-2577	1.012
Colorectal Cancer	CRC	SW620	Yes	RPMI 1640; 10% FCS; 0.05 mg/ml Gentamycin	NCI		15.698
Head and neck	HNSC	A-253	Yes	RPMI 1640; 10% FCS; 0.05 mg/ml Gentamycin	ATCC	HTB-41	18.691
Head and neck	HNSC	CAL-27	Yes	RPMI 1640; 10% FCS; 0.05 mg/ml Gentamycin	DSMZ	ACC 446	25.591
Head and neck	HNSC	CAL-33	Yes	RPMI 1640; 10% FCS; 0.05 mg/ml Gentamycin	DSMZ	ACC 447	25.101
Head and neck	HNSC	DETROIT562	Yes	RPMI 1640; 10% FCS; 0.05 mg/ml Gentamycin	ATCC	CCL-138	26.024
Head and neck	HNSC	FADU	Yes	RPMI 1640; 10% FCS; 0.05 mg/ml Gentamycin	ATCC	HTB-43	24.102
Head and neck	HNSC	HNXF_1853	Yes	RPMI 1640; 10% FCS; 0.05 mg/ml Gentamycin	CRL FR*		21.158
Head and neck	HNSC	HNXF_1859	Yes	RPMI 1640; 10% FCS; 0.05 mg/ml Gentamycin	CRL FR*		0.111
Head and neck	HNSC	RPMI-2650	Yes	RPMI 1640; 10% FCS; 0.05 mg/ml Gentamycin	DSMZ	ACC 287	0.91
Head and neck	HNSC	SNU-1076	Yes	RPMI 1640; 10% FCS; 0.05 mg/ml Gentamycin	KCLB	1076	17.27
Head and neck	HNSC	SNU-899	Yes	RPMI 1640; 10% FCS; 0.05 mg/ml Gentamycin	KCLB	899	23.29
Head and neck	HNSC	SW579	Yes	RPMI 1640; 10% FCS; 0.05 mg/ml Gentamycin	ATCC	HTB-107	12.813
Head and neck	HNSC	TT2609-C02	Yes	RPMI 1640; 10% FCS; 0.05 mg/ml Gentamycin	DSMZ	ACC 510	19.038
Kidney	Kidney	A498	Yes	RPMI 1640; 10% FCS; 0.05 mg/ml Gentamycin	NCI		1.046
Kidney	Kidney	ACHN	Yes	RPMI 1640; 10% FCS; 0.05 mg/ml Gentamycin	ECACC	88100508	0.535
Kidney	Kidney	CAKI-1	Yes	RPMI 1640; 10% FCS; 0.05 mg/ml Gentamycin	NCI		0.636
Kidney	Kidney	RXF_1183	Yes	RPMI 1640; 10% FCS; 0.05 mg/ml Gentamycin	CRL FR*		19.232
Kidney	Kidney	RXF_1220	Yes	RPMI 1640; 10% FCS; 0.05 mg/ml Gentamycin	CRL FR*		0.693
Kidney	Kidney	RXF_1781	Yes	RPMI 1640; 10% FCS; 0.05 mg/ml Gentamycin	CRL FR*		15.743
Kidney	Kidney	RXF_2282	Yes	RPMI 1640; 10% FCS; 0.05 mg/ml Gentamycin	CRL FR*		11.709
Kidney	Kidney	RXF_2516	Yes	RPMI 1640; 10% FCS; 0.05 mg/ml Gentamycin	CRL FR*		0.439

Kidney	Kidney	RXF_393	Yes	RPMI 1640; 10% FCS; 0.05 mg/ml Gentamycin	CRL FR*		15.142
Kidney	Kidney	RXF_486	Yes	RPMI 1640; 10% FCS; 0.05 mg/ml Gentamycin	CRL FR*		1.023
Kidney	Kidney	SN12C	Yes	RPMI 1640; 10% FCS; 0.05 mg/ml Gentamycin	NCI		21.055
Kidney	Kidney	TK10	No	RPMI 1640; 10% FCS; 0.05 mg/ml Gentamycin	NCI		26.399
Kidney	Kidney	UO-31	No	RPMI 1640; 10% FCS; 0.05 mg/ml Gentamycin	NCI		1.274
Kidney	Kidney	786-O	Yes	RPMI 1640; 10% FCS; 0.05 mg/ml Gentamycin	NCI		12.063
Leukaemia	Acute Lymphoblastic Leukaemia (LE_ALL)	CCRF-CEM	Yes	RPMI 1640; 10% FCS; 0.05 mg/ml Gentamycin	NCI		16.093
Leukaemia	Acute Lymphoblastic Leukaemia (LE_ALL)	CCRF-CEM-VCR	No	RPMI 1640; 10% FCS; 0.05 mg/ml Gentamycin	unknown		11.012
Leukaemia	Acute Lymphoblastic Leukaemia (LE_ALL)	JURKAT	Yes	RPMI 1640; 10% FCS; 0.05 mg/ml Gentamycin	DSMZ	ACC 282	11.949
Leukaemia	Acute Lymphoblastic Leukaemia (LE_ALL)	MOLT-3	No	RPMI 1640; 10% FCS; 0.05 mg/ml Gentamycin	DSMZ	ACC 84	0.631
Leukaemia	Acute Lymphoblastic Leukaemia (LE_ALL)	MOLT-4	Yes	RPMI 1640; 10% FCS; 0.05 mg/ml Gentamycin	NCI		0.306
Leukaemia	Acute Lymphoblastic Leukaemia (LE_ALL)	NALM-33	No	RPMI 1640; 10% FCS; 0.05 mg/ml Gentamycin	DSMZ	ACC 782	3.715
Leukaemia	Acute Lymphoblastic Leukaemia (LE_ALL)	RCH-ACV	Yes	RPMI; 20% FCS; 0.05 mg/ml Gentamycin	DSMZ	ACC 548	0.184
Leukaemia	Acute Lymphoblastic Leukaemia (LE_ALL)	SD1	No	RPMI 1640; 10% FCS; 0.05 mg/ml Gentamycin	DSMZ	ACC 366	0.153
Leukaemia	Acute Myeloid Leukaemia (LE_AML)	HL-60	Yes	RPMI 1640; 10% FCS; 0.05 mg/ml Gentamycin	DSMZ	ACC 366	13.367
Leukaemia	Acute Myeloid Leukaemia (LE_AML)	KG-1	Yes	RPMI 1640; 10% FCS; 0.05 mg/ml Gentamycin	DSMZ	ACC 14	12.414
Leukaemia	Acute Myeloid Leukaemia (LE_AML)	LEXFAM_2531	Yes	RPMI 1640; 10% FCS; 0.05 mg/ml Gentamycin	CRL FR*		0.847
Leukaemia	Acute Myeloid Leukaemia (LE_AML)	MOLM-13	Yes	RPMI 1640; 10% FCS; 0.05 mg/ml Gentamycin	DSMZ	ACC 554	0.677
Leukaemia	Acute Myeloid Leukaemia (LE_AML)	MV4-11	Yes	RPMI 1640; 10% FCS; 0.05 mg/ml Gentamycin	DSMZ	ACC 102	0.578
Leukaemia	Acute Myeloid Leukaemia (LE_AML)	NOMO-1	Yes	RPMI 1640; 10% FCS; 0.05 mg/ml Gentamycin	DSMZ	ACC 542	1.989
Leukaemia	Acute Myeloid Leukaemia (LE_AML)	OCI-AML2	Yes	RPMI 1640; 10% FCS; 0.05 mg/ml Gentamycin	DSMZ	ACC 99	11.536
Leukaemia	Acute Myeloid Leukaemia (LE_AML)	OCI-AML3	Yes	Alpha MEM; 20% FCS; 1% Gentamycin	DSMZ	ACC 582	0.123
Leukaemia	Acute Myeloid Leukaemia (LE_AML)	PL-21	Yes	RPMI 1640; 10% FCS; 0.05 mg/ml Gentamycin	DSMZ	ACC 536	15.662
Leukaemia	Acute Myeloid Leukaemia (LE_AML)	THP-1	Yes	RPMI 1640; 10% FCS; 0.05 mg/ml Gentamycin	DSMZ	ACC 16	12.425

Leukaemia	Acute Myeloid Leukaemia (LE_AML)	UOC-M1	No	McCoy; 10% FCS; 0.05 mg/ml Gentamycin	DSMZ	ACC 775	15.7
Leukaemia	Chronic Lymphocytic Leukaemia (LE_CLL)	MEC-1	Yes	RPMI 1640; 10% FCS; 0.05 mg/ml Gentamycin	DSMZ	ACC 497	9.373
Leukaemia	Chronic Myelogenous Leukaemia (LE_CML)	EM-2	Yes	RPMI 1640; 10% FCS; 0.05 mg/ml Gentamycin	DSMZ	ACC 135	15.555
Leukaemia	Chronic Myelogenous Leukaemia (LE_CML)	JURL-MK1	Yes	RPMI 1640; 10% FCS; 0.05 mg/ml Gentamycin	DSMZ	ACC 533	14.143
Leukaemia	Chronic Myelogenous Leukaemia (LE_CML)	K-562	Yes	RPMI 1640; 10% FCS; 0.05 mg/ml Gentamycin	NCI		12.227
Leukaemia	Chronic Myelogenous Leukaemia (LE_CML)	KCL-22	Yes	RPMI 1640; 10% FCS; 0.05 mg/ml Gentamycin	DSMZ	ACC 519	26.401
Leukaemia	Chronic Myelogenous Leukaemia (LE_CML)	MEG-01	Yes	RPMI 1640; 10% FCS; 0.05 mg/ml Gentamycin	DSMZ	ACC 364	10.932
Leukaemia	Chronic Myelogenous Leukaemia (LE_CML)	MOLM-1	No	RPMI; 20% FCS; 0.05 mg/ml Gentamycin	DSMZ	ACC 720	22.453
Leukaemia	Chronic Myelogenous Leukaemia (LE_CML)	MOLM-6	Yes	RPMI; 20% FCS; 0.05 mg/ml Gentamycin	DSMZ	ACC 611	10.704
Liver	Liver_cholangioma (Liver_chol)	EGI-1	No	MEM 10% FCS NEAA Glut Sod Pyr; 0.05 mg/ml Gentamycin	DSMZ	ACC 385	11.92
Liver	Liver_cholangioma (Liver_chol)	HEP-3B	No	RPMI 1640; 10% FCS; 0.05 mg/ml Gentamycin	DSMZ	ACC 93	14.321
Liver	Liver_cholangioma (Liver_chol)	LIXFC_575	No	RPMI 1640; 10% FCS; 0.05 mg/ml Gentamycin	CRL FR*		10.578
Liver	Liver_cholangioma (Liver_chol)	SK-HEP-1	Yes	RPMI 1640; 10% FCS; 0.05 mg/ml Gentamycin	DSMZ	ACC 141	2.955
Liver	Liver_cholangioma (Liver_chol)	TFK-1	No	RPMI 1640; 10% FCS; 0.05 mg/ml Gentamycin	DSMZ	ACC 344	14.236
Liver	Liver_hepatocellular (Liver_hep)	Hep-G2	Yes	RPMI 1640; 10% FCS; 0.05 mg/ml Gentamycin	DSMZ	ACC 180	0.358
Liver	Liver_hepatocellular (Liver_hep)	HLE	Yes	RPMI 1640; 10% FCS; 0.05 mg/ml Gentamycin	JCRB	JCRB0404	10.76
Liver	Liver_hepatocellular (Liver_hep)	JHH-4	Yes	RPMI 1640; 10% FCS; 0.05 mg/ml Gentamycin	JCRB	JCRB0435	9.767
Liver	Liver_hepatocellular (Liver_hep)	JHH-6	Yes	RPMI 1640; 10% FCS; 0.05 mg/ml Gentamycin	JCRB	JCRB1030	13.698
Liver	Liver_hepatocellular (Liver_hep)	SNU-398	Yes	RPMI 1640; 10% FCS; 0.05 mg/ml Gentamycin	KCLB	398	22.842
Liver	Liver_hepatocellular (Liver_hep)	SNU-423	Yes	RPMI 1640; 10% FCS; 0.05 mg/ml Gentamycin	KCLB	423	13.902
Liver	Liver_hepatocellular (Liver_hep)	SNU-449	Yes	RPMI 1640; 10% FCS; 0.05 mg/ml Gentamycin	KCLB	449	18.562
Liver	Liver_hepatocellular (Liver_hep)	SNU-475	Yes	RPMI 1640; 10% FCS; 0.05 mg/ml Gentamycin	KCLB	475	17.554
Liver	Liver_hepatocellular (Liver_hep)	SNU-739	No	RPMI 1640; 10% FCS; 0.05 mg/ml Gentamycin	KCLB	739	13.026
Liver	Liver_hepatocellular (Liver_hep)	SNU-761	Yes	RPMI 1640; 10% FCS; 0.05 mg/ml Gentamycin	KCLB	761	6.643

Liver	Liver_hepatocellular (Liver_hep)	SNU-878	Yes	RPMI 1640; 10% FCS; 0.05 mg/ml Gentamycin	KCLB	878	12.315
Lymphoma	Lymphoma_Burkitt (LY_Burkitt)	DAUDI	Yes	RPMI 1640; 10% FCS; 0.05 mg/ml Gentamycin	ATCC	CCL-213	0.368
Lymphoma	Lymphoma_Burkitt (LY_Burkitt)	DND-39	No	RPMI 1640; 10% FCS; 0.05 mg/ml Gentamycin	DSMZ	ACC 648	10.593
Lymphoma	Lymphoma_Burkitt (LY_Burkitt)	RAJI	Yes	RPMI 1640; 10% FCS; 0.05 mg/ml Gentamycin	ATCC	CCL-86	13.422
Lymphoma	Lymphoma_Burkitt (LY_Burkitt)	RAMOS	No	RPMI 1640; 10% FCS; 0.05 mg/ml Gentamycin	DSMZ	ACC 603	11.597
Lymphoma	Lymphoma_Diffuse Large B Cell (LY_DLBC)	SR	No	RPMI 1640; 10% FCS; 0.05 mg/ml Gentamycin	NCI		0.311
Lymphoma	Lymphoma_Diffuse Large B Cell (LY_DLBC)	SU-DHL-1	Yes	RPMI 1640; 10% FCS; 0.05 mg/ml Gentamycin	DSMZ	ACC 356	12.586
Lymphoma	Lymphoma_Diffuse Large B Cell (LY_DLBC)	SU-DHL-4	Yes	RPMI 1640; 10% FCS; 0.05 mg/ml Gentamycin	DSMZ	ACC 495	10.936
Lymphoma	Lymphoma_Diffuse Large B Cell (LY_DLBC)	U-937	Yes	RPMI 1640; 10% FCS; 0.05 mg/ml Gentamycin	DSMZ	ACC 5	14.174
Lymphoma	Lymphoma_Diffuse Large B Cell (LY_DLBC)	WSU-DLCL2	Yes	RPMI 1640; 10% FCS; 0.05 mg/ml Gentamycin	DSMZ	ACC 575	13.272
Lymphoma	Lymphoma_Hodgkin (LY_Hodgkin)	KM-H2	Yes	RPMI 1640; 10% FCS; 0.05 mg/ml Gentamycin	DSMZ	ACC 8	1.784
Lymphoma	Lymphoma_Multiple Myeloma (LY_MM)	EJM	Yes	IMDM; 20% FCS; 0.05 mg/ml Gentamycin	DSMZ	ACC 560	9.855
Lymphoma	Lymphoma_Multiple Myeloma (LY_MM)	IM-9	Yes	RPMI 1640; 10% FCS; 0.05 mg/ml Gentamycin	DSMZ	ACC 117	0.11
Lymphoma	Lymphoma_Multiple Myeloma (LY_MM)	L-363	Yes	RPMI 1640; 10% FCS; 0.05 mg/ml Gentamycin	DSMZ	ACC 49	11.554
Lymphoma	Lymphoma_Multiple Myeloma (LY_MM)	LP-1	Yes	RPMI 1640; 10% FCS; 0.05 mg/ml Gentamycin	DSMZ	ACC 41	10.528
Lymphoma	Lymphoma_Multiple Myeloma (LY_MM)	MM.1R	No	RPMI 1640; 10% FCS; 0.05 mg/ml Gentamycin	unknown		0.258
Lymphoma	Lymphoma_Multiple Myeloma (LY_MM)	MM.1S	Yes	RPMI 1640; 10% FCS; 0.05 mg/ml Gentamycin	unknown		0.242
Lymphoma	Lymphoma_Multiple Myeloma (LY_MM)	MOLP-2	Yes	RPMI; 20% FCS; 0.05 mg/ml Gentamycin + Glutamine	DSMZ	ACC 607	23.313
Lymphoma	Lymphoma_Multiple Myeloma (LY_MM)	NCI-H929	Yes	RPMI 1640; 10% FCS; 0.05 mg/ml Gentamycin	DSMZ	ACC 163	0.162
Lymphoma	Lymphoma_Multiple Myeloma (LY_MM)	OPM-2	Yes	RPMI 1640; 10% FCS; 0.05 mg/ml Gentamycin	DSMZ	ACC 50	6.586
Lymphoma	Lymphoma_Multiple Myeloma (LY_MM)	RPMI-8226	Yes	RPMI 1640; 10% FCS; 0.05 mg/ml Gentamycin	NCI		13.075
Lymphoma	Lymphoma_Multiple Myeloma (LY_MM)	U-266	No	RPMI 1640; 10% FCS; 0.05 mg/ml Gentamycin	DSMZ	ACC 9	14.856
Lymphoma	Lymphoma_unclass (LY_unclass)	HH	Yes	RPMI 1640; 10% FCS; 0.05 mg/ml Gentamycin	DSMZ	ACC 707	5.032
Lymphoma	Lymphoma_unclass (LY_unclass)	HUT-78	Yes	RPMI 1640; 10% FCS; 0.05 mg/ml Gentamycin	ECACC	88041901	12.932

Lymphoma	Lymphoma_unclass (LY_unclass)	MINO	Yes	RPMI 1640; 10% FCS; 0.05 mg/ml Gentamycin	DSMZ	ACC 687	7.753
Lymphoma	Lymphoma_unclass (LY_unclass)	OCI-LY18	No	RPMI 1640; 10% FCS; 0.05 mg/ml Gentamycin	DSMZ	ACC 699	1.426
Melanoma	Melanoma	MEXF_1341	Yes	RPMI 1640; 10% FCS; 0.05 mg/ml Gentamycin	CRL FR*		0.197
Melanoma	Melanoma	MEXF_1539	No	RPMI 1640; 10% FCS; 0.05 mg/ml Gentamycin	CRL FR*		0.447
Melanoma	Melanoma	MEXF_1737	No	RPMI 1640; 10% FCS; 0.05 mg/ml Gentamycin	CRL FR*		12.292
Melanoma	Melanoma	MEXF_1792	Yes	RPMI 1640; 10% FCS; 0.05 mg/ml Gentamycin	CRL FR*		0.593
Melanoma	Melanoma	MEXF_1829	Yes	RPMI 1640; 10% FCS; 0.05 mg/ml Gentamycin	CRL FR*		0.478
Melanoma	Melanoma	MEXF_2090	Yes	RPMI 1640; 10% FCS; 0.05 mg/ml Gentamycin	CRL FR*		13.451
Melanoma	Melanoma	MEXF_276	Yes	RPMI 1640; 10% FCS; 0.05 mg/ml Gentamycin	CRL FR*		0.923
Melanoma	Melanoma	MEXF_394	Yes	RPMI 1640; 10% FCS; 0.05 mg/ml Gentamycin	CRL FR*		3.664
Melanoma	Melanoma	MEXF_462	Yes	RPMI 1640; 10% FCS; 0.05 mg/ml Gentamycin	CRL FR*		14.124
Melanoma	Melanoma	MEXF_520	Yes	RPMI 1640; 10% FCS; 0.05 mg/ml Gentamycin	CRL FR*		1.392
Melanoma	Melanoma	MEXF_535	Yes	RPMI 1640; 10% FCS; 0.05 mg/ml Gentamycin	CRL FR*		19.336
Melanoma	Melanoma	MEXF_622	Yes	RPMI 1640; 10% FCS; 0.05 mg/ml Gentamycin	CRL FR*		0.442
Mesothelioma	Mesothelioma	H-MESO-1	No	RPMI 1640; 10% FCS; 0.05 mg/ml Gentamycin	NCI		27.958
Mesothelioma	Mesothelioma	MSTO-211H	Yes	RPMI 1640; 10% FCS; 0.05 mg/ml Gentamycin	DSMZ	ACC 390	0.649
Mesothelioma	Mesothelioma	PXF_1118	Yes	RPMI 1640; 10% FCS; 0.05 mg/ml Gentamycin	CRL FR*		0.69
Mesothelioma	Mesothelioma	PXF_1752	Yes	RPMI 1640; 10% FCS; 0.05 mg/ml Gentamycin	CRL FR*		15.578
Mesothelioma	Mesothelioma	PXF_698	Yes	RPMI 1640; 10% FCS; 0.05 mg/ml Gentamycin	CRL FR*		16.194
Non-Small Cell Lung	Adenocarcinoma (NSCL_ad)	A427	No	RPMI 1640; 10% FCS; 0.05 mg/ml Gentamycin	ATCC	HTB-53	5.223
Non-Small Cell Lung	Adenocarcinoma (NSCL_ad)	LXFA_1647	Yes	RPMI 1640; 10% FCS; 0.05 mg/ml Gentamycin	CRL FR*		15.762
Non-Small Cell Lung	Adenocarcinoma (NSCL_ad)	LXFA_289	Yes	RPMI 1640; 10% FCS; 0.05 mg/ml Gentamycin	CRL FR*		11.021
Non-Small Cell Lung	Adenocarcinoma (NSCL_ad)	LXFA_526	Yes	RPMI 1640; 10% FCS; 0.05 mg/ml Gentamycin	CRL FR*		15.636
Non-Small Cell Lung	Adenocarcinoma (NSCL_ad)	LXFA_586	Yes	RPMI 1640; 10% FCS; 0.05 mg/ml Gentamycin	CRL FR*		2.338

Non-Small Cell Lung	Adenocarcinoma (NSCL_ad)	LXFA_623	Yes	RPMI 1640; 10% FCS; 0.05 mg/ml Gentamycin	CRL FR*		18.672
Non-Small Cell Lung	Adenocarcinoma (NSCL_ad)	LXFA_629	Yes	RPMI 1640; 10% FCS; 0.05 mg/ml Gentamycin	CRL FR*		13.67
Non-Small Cell Lung	Adenocarcinoma (NSCL_ad)	LXFA_677	Yes	RPMI 1640; 10% FCS; 0.05 mg/ml Gentamycin	CRL FR*		18.777
Non-Small Cell Lung	Adenocarcinoma (NSCL_ad)	LXFA_737	Yes	RPMI 1640; 10% FCS; 0.05 mg/ml Gentamycin	CRL FR*		2.017
Non-Small Cell Lung	Adenocarcinoma (NSCL_ad)	LXFA_923	Yes	RPMI 1640; 10% FCS; 0.05 mg/ml Gentamycin	CRL FR*		5.428
Non-Small Cell Lung	Adenocarcinoma (NSCL_ad)	LXFA_983	Yes	RPMI 1640; 10% FCS; 0.05 mg/ml Gentamycin	CRL FR*		17.415
Non-Small Cell Lung	Adenocarcinoma (NSCL_ad)	NCI-H1975	Yes	RPMI 1640; 10% FCS; 0.05 mg/ml Gentamycin	ATCC	CRL-5908	14.319
Non-Small Cell Lung	Adenocarcinoma (NSCL_ad)	NCI-H322M	No	RPMI 1640; 10% FCS; 0.05 mg/ml Gentamycin	NCI		20.987
Non-Small Cell Lung	Adenocarcinoma (NSCL_ad)	NCI-H441	Yes	RPMI 1640; 10% FCS; 0.05 mg/ml Gentamycin	NCI		16.887
Non-Small Cell Lung	Adenocarcinoma (NSCL_ad)	PC-9	No	RPMI 1640; 10% FCS; 0.05 mg/ml Gentamycin	ECACC	90071810	15.841
Non-Small Cell Lung	epidermoid (NSCL_ep)	Calu-1	Yes	McCoy; 10% FCS; 0.05 mg/ml Gentamycin	ATCC	HTB-54	29.459
Non-Small Cell Lung	epidermoid (NSCL_ep)	LOU-NH91	Yes	RPMI; 20% FCS; 0.05 mg/ml Gentamycin	DSMZ	ACC 393	16.107
Non-Small Cell Lung	epidermoid (NSCL_ep)	LXFE_2478	Yes	RPMI 1640; 10% FCS; 0.05 mg/ml Gentamycin	CRL FR*		23.136
Non-Small Cell Lung	epidermoid (NSCL_ep)	LXFE_66	Yes	RPMI 1640; 10% FCS; 0.05 mg/ml Gentamycin	CRL FR*		24.595
Non-Small Cell Lung	epidermoid (NSCL_ep)	SK-MES-1	Yes	RPMI 1640; 10% FCS; 0.05 mg/ml Gentamycin	DSMZ	ACC 353	13.901
Non-Small Cell Lung	Large cells (NSCL_large)	LXFL_1072	Yes	RPMI 1640; 10% FCS; 0.05 mg/ml Gentamycin	CRL FR*		26.578
Non-Small Cell Lung	Large cells (NSCL_large)	LXFL_1121	Yes	RPMI 1640; 10% FCS; 0.05 mg/ml Gentamycin	CRL FR*		18.295
Non-Small Cell Lung	Large cells (NSCL_large)	LXFL_1674	Yes	RPMI 1640; 10% FCS; 0.05 mg/ml Gentamycin	CRL FR*		11.414
Non-Small Cell Lung	Large cells (NSCL_large)	LXFL_430	Yes	RPMI 1640; 10% FCS; 0.05 mg/ml Gentamycin	CRL FR*		15.359
Non-Small Cell Lung	Large cells (NSCL_large)	LXFL_529	Yes	RPMI 1640; 10% FCS; 0.05 mg/ml Gentamycin	CRL FR*		14.726
Non-Small Cell Lung	Large cells (NSCL_large)	NCI-H1299	Yes	RPMI 1640; 10% FCS; 0.05 mg/ml Gentamycin	ATCC	CRL-5803	16.647
Non-Small Cell Lung	Large cells (NSCL_large)	NCI-H460	Yes	RPMI 1640; 10% FCS; 0.05 mg/ml Gentamycin	NCI		1.275
Non-Small Cell Lung	unclassified (NSCL_unclass)	NCI-H522	Yes	RPMI 1640; 10% FCS; 0.05 mg/ml Gentamycin	NCI		10.766
Oesophagus	Oesophagus	KYSE-150	Yes	50%RPMI; 50% Ham'sF12; 5% FCS; 0.05 mg/ml Gentamycin	DSMZ	ACC 375	11.635

Oesophagus	Oesophagus	KYSE-180	Yes	RPMI 1640; 10% FCS; 0.05 mg/ml Gentamycin	DSMZ	ACC 379	18.147
Oesophagus	Oesophagus	KYSE-520	Yes	RPMI 1640; 10% FCS; 0.05 mg/ml Gentamycin	DSMZ	ACC 371	11.383
Oesophagus	Oesophagus	KYSE-70	Yes	RPMI 1640; 10% FCS; 0.05 mg/ml Gentamycin	DSMZ	ACC 363	19.258
Oesophagus	Oesophagus	OE21	Yes	RPMI 1640; 10% FCS; 0.05 mg/ml Gentamycin	Public Health England		18.809
Ovary	Ovary	A2780	Yes	RPMI 1640; 10% FCS; 0.05 mg/ml Gentamycin	NCI		11.623
Ovary	Ovary	EFO-21	Yes	RPMI; 20% FCS; 0.05 mg/ml Gentamycin +Glutamine	DSMZ	ACC 235	24.125
Ovary	Ovary	EFO-27	Yes	RPMI 20% FBS, 2mM L-Glutamine, 1xMEM non-essential Amino acids, 1mM Sodiumpyruvat; 0.05 mg/ml Gentamycin	DSMZ	ACC 191	24.785
Ovary	Ovary	IGROV-1	Yes	RPMI 1640; 10% FCS; 0.05 mg/ml Gentamycin	NCI		6.908
Ovary	Ovary	OVCAR-3	No	RPMI 1640; 10% FCS; 0.05 mg/ml Gentamycin	NCI		16.782
Ovary	Ovary	OVCAR-4	Yes	RPMI 1640; 10% FCS; 0.05 mg/ml Gentamycin	NCI		15.998
Ovary	Ovary	OVCAR-5	Yes	RPMI 1640; 10% FCS; 0.05 mg/ml Gentamycin	NCI		30.593
Ovary	Ovary	OVCAR-8	Yes	RPMI 1640; 10% FCS; 0.05 mg/ml Gentamycin	NCI		18.763
Ovary	Ovary	OVXF_1023	Yes	RPMI 1640; 10% FCS; 0.05 mg/ml Gentamycin	CRL FR*		21.961
Ovary	Ovary	OVXF_899	Yes	RPMI 1640; 10% FCS; 0.05 mg/ml Gentamycin	CRL FR*		1.093
Ovary	Ovary	SK-OV-3	Yes	RPMI 1640; 10% FCS; 0.05 mg/ml Gentamycin	NCI		20.586
Pancreas	Pancreas	HPAC	Yes	RPMI 1640; 10% FCS; 0.05 mg/ml Gentamycin	ATCC	CRL-2119	13.817
Pancreas	Pancreas	HPAF-II	Yes	EMEM; 10% FCS; 0.05 mg/ml Gentamycin	ATCC	CRL-1997	10.812
Pancreas	Pancreas	HUP-T3	Yes	RPMI 1640; 10% FCS; 0.05 mg/ml Gentamycin	DSMZ	ACC 259	12.231
Pancreas	Pancreas	MIA-PaCa-2	Yes	RPMI 1640; 10% FCS; 0.05 mg/ml Gentamycin	ATCC	CRL-1420	11.426
Pancreas	Pancreas	PANC-1	Yes	RPMI 1640; 10% FCS; 0.05 mg/ml Gentamycin	CLS	300228	17.12
Pancreas	Pancreas	PA-TU-8902	Yes	RPMI 1640; 10% FCS; 0.05 mg/ml Gentamycin	DSMZ	ACC 179	19.935
Pancreas	Pancreas	PA-TU-8988T	Yes	RPMI 1640; 10% FCS; 0.05 mg/ml Gentamycin	DSMZ	ACC 162	12.559
Pancreas	Pancreas	PAXF_1657	Yes	RPMI 1640; 10% FCS; 0.05 mg/ml Gentamycin	CRL FR*		21.126

Pancreas	Pancreas	PAXF_1986	Yes	RPMI 1640; 10% FCS; 0.05 mg/ml Gentamycin	CRL FR*		20.569
Pancreas	Pancreas	PAXF_1997	Yes	RPMI 1640; 10% FCS; 0.05 mg/ml Gentamycin	CRL FR*		20.638
Pancreas	Pancreas	PAXF_1998	Yes	RPMI 1640; 10% FCS; 0.05 mg/ml Gentamycin	CRL FR*		28.64
Pancreas	Pancreas	PAXF_2005	Yes	RPMI 1640; 10% FCS; 0.05 mg/ml Gentamycin	CRL FR*		14.571
Pancreas	Pancreas	PAXF_2035	Yes	RPMI 1640; 10% FCS; 0.05 mg/ml Gentamycin	CRL FR*		27.317
Pancreas	Pancreas	PAXF_2059	Yes	RPMI 1640; 10% FCS; 0.05 mg/ml Gentamycin	CRL FR*		11.852
Pancreas	Pancreas	PAXF_546	Yes	RPMI 1640; 10% FCS; 0.05 mg/ml Gentamycin	CRL FR*		16.073
Prostate	Prostate	Lncap	No	RPMI 1640; 10% FCS; 0.05 mg/ml Gentamycin	ATCC	CRL-1740	0.169
Prostate	Prostate	22RV1	Yes	RPMI 1640; 10% FCS; 0.05 mg/ml Gentamycin	DSMZ	ACC 438	1.147
Prostate	Prostate	DU-145	Yes	RPMI 1640; 10% FCS; 0.05 mg/ml Gentamycin	NCI		12.952
Prostate	Prostate	PC-3M	Yes	RPMI 1640; 10% FCS; 0.05 mg/ml Gentamycin	NCI		13.481
Prostate	Prostate	VCAP	Yes	DMEM; 10% FCS; 0.05 mg/ml Gentamycin	ATCC	CRL-2876	14.603
Sarcoma	Sarcoma Ewing (SA_Ewing)	RD-ES	Yes	RPMI 1640; 10% FCS; 0.05 mg/ml Gentamycin	DSMZ	ACC 260	12.139
Sarcoma	osteosarcoma (SA_osteo)	Saos-2	No	RPMI 1640; 10% FCS; 0.05 mg/ml Gentamycin	DSMZ	ACC 243	16.407
Sarcoma	osteosarcoma (SA_osteo)	SXFO_678	Yes	RPMI 1640; 10% FCS; 0.05 mg/ml Gentamycin	CRL FR*		5.037
Sarcoma	osteosarcoma (SA_osteo)	U-2-OS	Yes	McCoy; 10% FCS; 0.05 mg/ml Gentamycin	DSMZ	ACC 785	0.674
Sarcoma	Sarcoma soft tissue (SA_soft t)	A204	Yes	McCoy; 10% FCS; 0.05 mg/ml Gentamycin	DSMZ	ACC 250	5.269
Sarcoma	Sarcoma soft tissue (SA_soft t)	Hs 729	Yes	RPMI 1640; 10% FCS; 0.05 mg/ml Gentamycin	ATCC	HTB-153	18.817
Sarcoma	Sarcoma soft tissue (SA_soft t)	HT-1080	Yes	RPMI 1640; 10% FCS; 0.05 mg/ml Gentamycin	ATCC	CCL-121	0.984
Sarcoma	Sarcoma soft tissue (SA_soft t)	RH-30	Yes	RPMI 1640; 10% FCS; 0.05 mg/ml Gentamycin	DSMZ	ACC 489	15.033
Sarcoma	Sarcoma soft tissue (SA_soft t)	RH-41	Yes	RPMI 1640; 10% FCS; 0.05 mg/ml Gentamycin	DSMZ	ACC 592	11.297
Sarcoma	Sarcoma soft tissue (SA_soft t)	SK-LMS-1	Yes	RPMI 1640; 10% FCS; 0.05 mg/ml Gentamycin	ATCC	HTB-88	29.61
Sarcoma	Sarcoma soft tissue (SA_soft t)	SXFS_1301	Yes	RPMI 1640; 10% FCS; 0.05 mg/ml Gentamycin	CRL FR*		10.712
Sarcoma	Sarcoma soft tissue (SA_soft t)	TE671	No	RPMI 1640; 10% FCS; 0.05 mg/ml Gentamycin	DSMZ	ACC 263	27.324

Sarcoma	Sarcoma soft tissue (SA_soft t)	U-2197	No	EMEM; 20% FCS; 0.05 mg/ml Gentamycin	DSMZ	ACC 406	11.186
Small Cell Lung Cancer	SCLC	DMS-273	Yes	RPMI 1640; 10% FCS; 0.05 mg/ml Gentamycin	NCI		17.247
Small Cell Lung Cancer	SCLC	H69AR	Yes	RPMI 20% FCS; 1% Sodiumpyruvat; 0.05 mg/ml Gentamycin	ATCC	CRL-11351	13.731
Small Cell Lung Cancer	SCLC	HCC33	Yes	RPMI; 20% FCS; 0.05 mg/ml Gentamycin	DSMZ	ACC 487	14.439
Small Cell Lung Cancer	SCLC	NCI-H1184	Yes	RPMI; 20% FCS; 0.05 mg/ml Gentamycin	DSMZ	ACC 502	30
Small Cell Lung Cancer	SCLC	NCI-H2171	Yes	RPMI; 20% FCS; 0.05 mg/ml Gentamycin	DSMZ	ACC 544	30
Small Cell Lung Cancer	SCLC	NCI-H345	Yes	RPMI 1640; 10% FCS; 0.05 mg/ml Gentamycin	NCI		30
Small Cell Lung Cancer	SCLC	NCI-H69	Yes	RPMI 1640; 10% FCS; 0.05 mg/ml Gentamycin	ATCC	HTB-119	0.258
Small Cell Lung Cancer	SCLC	NCI-H727	Yes	RPMI 1640; 10% FCS; 0.05 mg/ml Gentamycin	NCI		12.962
Small Cell Lung Cancer	SCLC	NCI-H82	Yes	RPMI 1640; 10% FCS; 0.05 mg/ml Gentamycin	DSMZ	ACC 556	16.158
Small Cell Lung Cancer	SCLC	SCLC-21H	Yes	DMEM; 10% FCS; 0.05 mg/ml Gentamycin	DSMZ	ACC 372	16.357
Skin	Skin	A-431	Yes	McCoy; 10% FCS; 0.05 mg/ml Gentamycin	ATCC	CRL-1555	16.724
Stomach	Stomach	FU97	Yes	RPMI 1640; 10% FCS; 0.05 mg/ml Gentamycin	JCRB	JCRB1074	12.093
Stomach	Stomach	GXA_3011	Yes	RPMI 1640; 10% FCS; 0.05 mg/ml Gentamycin	CRL FR*		18.971
Stomach	Stomach	GXA_3013	Yes	RPMI 1640; 10% FCS; 0.05 mg/ml Gentamycin	CRL FR*		16.599
Stomach	Stomach	GXA_3023	Yes	RPMI 1640; 10% FCS; 0.05 mg/ml Gentamycin	CRL FR*		14.279
Stomach	Stomach	GXA_3067	Yes	RPMI 1640; 10% FCS; 0.05 mg/ml Gentamycin	CRL FR*		12.963
Stomach	Stomach	GXF_1172	Yes	RPMI 1640; 10% FCS; 0.05 mg/ml Gentamycin	CRL FR*		0.519
Stomach	Stomach	GXF_23132	Yes	RPMI 1640; 10% FCS; 0.05 mg/ml Gentamycin	DSMZ	ACC 201	12.512
Stomach	Stomach	GXF_251	Yes	RPMI 1640; 10% FCS; 0.05 mg/ml Gentamycin	CRL FR*		14.268
Stomach	Stomach	IM95	Yes	RPMI 1640; 10% FCS; 0.05 mg/ml Gentamycin	JCRB	JCRB1075.0	4.056
Stomach	Stomach	MKN1	Yes	RPMI 1640; 10% FCS; 0.05 mg/ml Gentamycin	JCRB	JCRB0252	13.69
Stomach	Stomach	MKN45	Yes	RPMI 1640; 10% FCS; 0.05 mg/ml Gentamycin	JCRB	JCRB0254	0.725
Stomach	Stomach	MKN7	Yes	RPMI 1640; 10% FCS; 0.05 mg/ml Gentamycin	JCRB	JCRB1025	22.834

Stomach	Stomach	NUGC-4	Yes	RPMI 1640; 10% FCS; 0.05 mg/ml Gentamycin	JCRB	JCRB0834	1.794
Stomach	Stomach	OCUM-1	Yes	RPMI 1640; 10% FCS; 0.05 mg/ml Gentamycin	JCRB	JCRB0192	0.216
Stomach	Stomach	SK-GT-2	No	EMEM; 10% FCS; 0.05 mg/ml Gentamycin	DSMZ	ACC 702	24.55
Stomach	Stomach	SNU-16	Yes	RPMI 1640; 10% FCS; 0.05 mg/ml Gentamycin	KCLB	16	21.167
Uterus	Uterus_cervix	DoTc2-4510	Yes	RPMI 1640; 10% FCS; 0.05 mg/ml Gentamycin	ATCC	CRL-7920	17.713
Uterus	Uterus_cervix	HeLa	Yes	RPMI 1640; 10% FCS; 0.05 mg/ml Gentamycin	NCI		17.605
Uterus	Uterus_cervix	SiHa	No	EMEM; 10% FCS; 0.05 mg/ml Gentamycin	ATCC	HTB-35	13.915
Uterus	Uterus_cervix	SISO	No	RPMI 1640; 10% FCS; 0.05 mg/ml Gentamycin	DSMZ	ACC 327	12.712
Uterus	Uterus_cervix	SW756	Yes	RPMI 1640; 10% FCS; 0.05 mg/ml Gentamycin	ATCC	CRL-10302	20.074
Uterus	Uterus_endometrium (Uterus_endo)	EFE-184	Yes	RPMI 1640; 10% FCS; 0.05 mg/ml Gentamycin	DSMZ	ACC 230	20.511
Uterus	Uterus_endometrium (Uterus_endo)	HEC-1-A	Yes	RPMI 1640; 10% FCS; 0.05 mg/ml Gentamycin	ATCC	HTB-112	16.519
Uterus	Uterus_endometrium (Uterus_endo)	KLE	Yes	RPMI 1640; 10% FCS; 0.05 mg/ml Gentamycin	ATCC	CRL-1622	15.992
Uterus	Uterus_endometrium (Uterus_endo)	MFE-319	Yes	RPMI 1640; 10% FCS; 0.05 mg/ml Gentamycin	DSMZ	ACC 423	16.773
Uterus	Uterus_endometrium (Uterus_endo)	SK-UT-1B	No	RPMI 1640; 10% FCS; 0.05 mg/ml Gentamycin	ATCC	HTB-115	12.526
Uterus	Uterus_endometrium (Uterus_endo)	UXF_1138	Yes	RPMI 1640; 10% FCS; 0.05 mg/ml Gentamycin	CRL FR*		15.103

*** CRL FR: Charles River Laboratories Freiburg. Cell lines established from patient-derived xenograft (PDX) developed by Charles River Laboratories Freiburg**

**** Molecular data: Whole Exome + Affymetrix SNP6.0 array + Affymetrix HGU133 Plus 2.0 GeneChip arrays profiles**

Supplementary Table 2. Distribution of the 274 CLs across tumour (sub)types and MI-773 drug response classes.

	Highly sensitive (Hs) < 1 µM	Intermediate sensitive (Is) [1,10[µM	Resistant ≥ 10 µM	Total	% Sensitive (Hs + Is)
**CLs from solid tumours**					
Melanoma	6	2	4	12	67
Kidney	4	3	7	14	50
Mesothelioma	2	0	3	5	40
Prostate	1	1	3	5	40
CNS	1	2	6	9	33
Sarcoma	2	2	9	13	31
Stomach	3	2	11	16	31
Breast	2	2	11	15	27
Bladder	0	2	6	8	25
CRC	1	3	12	16	25
Liver	1	3	12	16	25
NSCLC	0	5	23	28	18
Ovary	0	2	9	11	18
HNSC	2	0	10	12	17
SCLC	1	0	9	10	10
Skin	0	0	1	1	0
Oesophagus	0	0	5	5	0
Pancreas	0	0	15	15	0
Uterus	0	0	11	11	0
**Total**	**26**	**29**	**167**	**222**	
**CLs from haematological cancers**					
LY_MM	4	2	5	11	55
LY_Burkitt	1	0	3	4	25
LY_DLBC	1	0	4	5	20
LY_Hodgkin	0	1	0	1	100
LY_unclass	0	3	1	4	75
LE_CLL	0	1	0	1	100
LE_ALL	4	1	3	8	62
LE_AML	4	1	6	11	45
LE_CML	0	0	7	7	0
**Total**	**14**	**9**	**29**	**52**	
**Total**	**40**	**38**	**196**	**274**	

Supplementary Table 3. Correlation of the anti-cancer agents in the 4HF Biotec, GDSC1 and GDSC2 databases with MI-773 Abs IC50 by COMPARE analysis.

4HF Biotec (a)

**Compound**	**Target**	**CLs (*n*=)**	**ρ (Spearman test)**	**P-value**
Nutlin-3a	p53/MDM2	273	0.83	2.72E-71
RG-7112	MDM2	243	0.64	2.33E-29
GNE-7915	LRRK2	51	0.48	4.12E-04
YH239-EE	p53/MDM2	50	0.48	4.90E-04
RN 4188/MG 011	CK1	118	0.47	7.69E-08
CHIR-98014	GSK3-alpha/beta	51	0.42	2.20E-03
AZD1080	GSK3-alpha/beta	50	0.42	2.62E-03
BI-847325	MEK1/2, Aurora A/C	273	0.41	1.15E-12
TP-0903	AXL	255	0.41	7.05E-12
CHIR-99021	GSK-3a/ß	41	0.41	8.18E-03
RN 4375/TS 005	CK1	80	0.4	2.96E-04
RN 4373/TS 004	CK1	50	0.4	3.80E-03
AZD6738	ATR	51	0.39	4.69E-03
RAF265	B-Raf, VEGFR2	50	0.39	4.87E-03
Dabrafenib	B-Raf, C-Raf	41	0.39	1.19E-02
Pomalidomide	TNF-alpha	29	0.39	3.43E-02
LY2835219 sulphate	CDK4/6	211	0.38	1.72E-08
RN 4381/TS 001	CK1	210	0.36	6.66E-08
LN2000	p38 MAPK	151	0.36	5.95E-06
PD0166285	Wee1, Chk1	30	0.36	5.19E-02
LY2090314	GSK-3a/ß	273	0.35	4.69E-09
Panobinostat, free base	HDAC1; HDAC2; HDAC3; HDAC6; HDAC8	127	0.35	5.48E-05
LN 808/SK 797	p38 MAPK	117	0.35	8.68E-05
LRRK2-IN-1	LRRK2	80	0.35	1.39E-03
MS436	BRD4	50	0.35	1.24E-02
LY2109761	TGF-beta RI/II	51	0.35	1.25E-02
KS 428/LN 1488	JNK3	50	0.35	1.29E-02
VE-822	ATR	272	0.34	7.59E-09
LN 789/SK 564	p38 MAPK	133	0.34	5.65E-05
SB525334	TGF-beta RI	58	0.34	8.83E-03
GSK1324726A	BRD2, BRD3, BRD4	273	0.33	2.63E-08
LN 1222/AD 736	p38 MAPK	243	0.33	1.25E-07
AMG925	Flt-3, CDK4	147	0.33	4.22E-05
LN2183/HW201	p38 MAPK	117	0.33	3.47E-04
BIO	GSK3, JAK3	51	0.33	1.75E-02
MGCD-265	c-Met, VEGFR1/2/3	51	0.33	1.76E-02
CPI-203	BRD4	272	0.32	4.44E-08
LN 2119/FM-315	JAK3	148	0.31	1.17E-04
Palbociclib HCl	CDK4/6	140	0.31	1.59E-04
LN 2184/HW202	p38 MAPK	51	0.31	2.52E-02
SB216763	GSK3	41	0.31	5.18E-02
TH287	MTH1	264	0.3	7.41E-07

Idelalisib	PI3K	96	0.3	2.85E-03
Sirtinol	SIRT1/2	51	0.3	2.98E-02
NPS-1034	c-Met, AXL	51	0.3	3.01E-02
GSK503	EZH2	50	0.3	3.71E-02
NMS-E973	HSP90	30	0.3	1.05E-01
UNC-2025	Mer, Flt-3	29	0.3	1.20E-01
LN 2148/GM 302	EGFR	13	0.3	3.15E-01
LN 2206/FM-375	JAK3	241	0.29	5.27E-06
LN2015	p38 MAPK	210	0.29	2.21E-05
LN 1476/FS 548	p38 MAPK	116	0.29	1.54E-03
Flavopiridol HCl	CDK2/4/7	78	0.29	1.09E-02
AMG-208	c-Met	51	0.29	3.62E-02
LN 1066/SK 846	p38 MAPK	50	0.29	3.99E-02
TG101348	JAK2	51	0.29	4.10E-02
H-101	CDK1/4/9	30	0.29	1.19E-01
BMS-265246	CDK1/2	272	0.28	2.01E-06
PIT0106006/TK01020	JNK3	80	0.28	1.05E-02
Dovitinib, free base	Multikinase inhibitor (Flt-3, c-Kit, FGFR1/3, VEGFR1/2/3/4)	41	0.28	7.67E-02
ARQ 621	Eg5	30	0.28	1.34E-01
OTX015, free base	BRD2, BRD3, BRD4	152	0.26	1.44E-03
SP 600125	JNK3	80	0.26	2.06E-02
PLX-4720	B-Raf	59	0.25	5.74E-02
LN 672/JH 126	p38 MAPK	51	0.25	8.00E-02
GDC-0879	B-Raf	83	0.24	2.81E-02
Carfilzomib	Proteasome	272	0.23	1.11E-04
FLLL32	JAK2, STAT3	80	0.23	4.18E-02
Crizotinib, free base	c-Met, ALK	69	0.23	6.01E-02
SNS-032	CDK2/7/9	50	0.23	1.02E-01
AMG-458	c-Met	49	0.23	1.18E-01
Cabozantinib	Multikinase (VEGFR2, c-Met, Ret, c-Kit, Flt-3, Tie2, AXL)	41	0.23	1.52E-01
Taselisib	PI3K	273	0.22	3.37E-04
LCL-161	IAP	116	0.22	1.97E-02
Afuresertib	AKT1/2/3	51	0.22	1.23E-01
LN 1891/MG-2-631	JAK3	113	0.21	2.29E-02
LEE011	CDK4/6	117	0.21	2.30E-02
Pelitinib	EGFR	79	0.21	6.05E-02
LN 986/SK 824	p38 MAPK	51	0.21	1.39E-01
ABT-737	Bcl-xL, Bcl-2 and Bcl-w	41	0.21	1.84E-01
SGI-7079	AXL	29	0.21	2.73E-01
Ceritinib	ALK	108	0.2	3.40E-02
AZD5438	CDK1/2/9	51	0.2	1.51E-01
JNJ-38877605	c-Met	52	0.2	1.53E-01
Cyclo(RGDyK)	aVb3 integrin	102	0.19	5.69E-02
Rociletinib	EGFR	101	0.18	6.46E-02
MK-2206 dihydrochloride	AKt1/2/3	57	0.18	1.76E-01

Pacritinib	JAK2	50	0.18	2.13E-01
LN 1215/AD 722	p38 MAPK	80	0.17	1.30E-01
CEP-32496	B-Raf, C-Raf	50	0.17	2.24E-01
AT7519	CDK1/2/4/6/9	50	0.17	2.49E-01
MK-8745	Aurora A	29	0.17	3.88E-01
SK 288/LN 778	p38 MAPK	117	0.16	9.33E-02
Pazopanib, free base	Multikinase (VEGFR1/2/3, PDGFR-alpha/beta, c-Kit)	109	0.16	1.07E-01
SB590885	B-Raf	58	0.16	2.20E-01
Golvatinib	c-Met, VEGFR2	51	0.16	2.49E-01
TIC10	AKT, ERK	50	0.16	2.62E-01
Oprozomib	Proteasome	50	0.16	2.63E-01
TAK-632	B-Raf, C-Raf	51	0.16	2.65E-01
Erlotinib HCl	EGFR	145	0.14	9.90E-02
PFI-1	BRD4	50	0.14	3.30E-01
Apabetalone	BRD4	49	0.14	3.42E-01
GS-0387	JAK1 / JAK2	51	0.13	3.45E-01
LN 802/SK 676	p38 MAPK	51	0.13	3.64E-01
Ipatasertib	AKT1/2/3	51	0.13	3.69E-01
Sabutoclax	Bcl-xL, Mcl-1, Bfl-1	272	0.12	5.79E-02
(+)-JQ1	BRD4	117	0.12	2.13E-01
Doxorubicin HCl	DNA intercalating, TOP2A	96	0.12	2.40E-01
PF-04217903	c-Met	87	0.12	2.50E-01
GSK2578215A	LRRK2	51	0.12	3.86E-01
Silmitasertib	CK2	51	0.12	4.16E-01
JNJ-7706621	CDK1/2, Aurora AB/B	51	0.12	4.18E-01
I-BET151	BRD2, BRD3, BRD4	117	0.11	2.28E-01
Decernotinib	JAK3	51	0.11	4.28E-01
TWS119	GSK3-beta	50	0.11	4.52E-01
MK-2461	c-Met	60	0.1	4.47E-01
Ixazomib	Proteasome	58	0.1	4.56E-01
Ro3280	PLK1	30	0.1	5.92E-01
RO5126766	B-Raf, MEK	183	0.09	2.25E-01
Canertinib dihydrochloride	EGFR, HER2/3/4	52	0.09	5.11E-01
PIT0106005	JNK3	49	0.09	5.56E-01
XAV-939	TNKS	41	0.09	5.61E-01
AT-101	Bcl-2, Bcl-xL and Mcl-1	38	0.09	5.83E-01
Ibrutinib	BTK	211	0.08	2.43E-01
HA14-1	Bcl-2	50	0.08	5.71E-01
AZD1480	JAK2	50	0.08	5.71E-01
SB415286	GSK3-alpha/beta	51	0.08	5.80E-01
AZD5363	AKT	41	0.08	6.29E-01
Tideglusib	GSK3-beta	51	0.07	6.43E-01
LN2056/GM-258	EGFR	32	0.07	7.22E-01
GDC-0623	MEK1	259	0.06	3.05E-01
AZD3463	ALK	50	0.06	6.75E-01
NMS-P937	PK1	29	0.06	7.62E-01

Lapatinib, free base	EGFR, HER2	141	0.05	5.84E-01
D4476	CK1	50	0.05	7.13E-01
SBE 13 HCl	PLK1	30	0.05	7.85E-01
ONX-0914	Proteasome	116	0.04	6.86E-01
VR23	Proteasome	51	0.04	7.81E-01
Ruxolitinib	JAK1/2	50	0.04	7.91E-01
LDC1267	AXL, Mer, Tyro3	29	0.04	8.25E-01
PF-477736	Chk1	27	0.04	8.61E-01
BV-6	IAP	211	0.03	6.87E-01
TW-37	Bcl-xL, Mcl-1	51	0.03	8.16E-01
TDZD-8	GSK-3ß	26	0.03	8.71E-01
VER155008	HSP70	30	0.03	8.92E-01
Paclitaxel	Beta subunit of Tubulin	117	0.02	8.29E-01
Gefitinib	EGFR	125	0.02	8.67E-01
LN2057	EGFR	32	0.02	9.29E-01
LN 2297	EGFR	14	0.02	9.58E-01
CH5138303	HSP90	30	0.01	9.50E-01
LN2147/GM 352	EGFR	32	-0.01	9.46E-01
Afatinib, free base	EGFR, HER2	79	-0.01	9.65E-01
ZM 336372	C-Raf	50	-0.02	8.65E-01
VER-49009	HSP90	46	-0.02	9.07E-01
LN 1220/AD 725	p38 MAPK	48	-0.03	8.37E-01
Alectinib	ALK	42	-0.04	8.13E-01
SD-208	TGF-beta RI	30	-0.04	8.34E-01
INC280, free base	c-Met	41	-0.05	7.44E-01
Obatoclax mesylate	Bcl-2	41	-0.07	6.84E-01
Peficitinib	JAK	50	-0.08	5.63E-01
WZ 4002/LN 2125	EGFR	32	-0.08	6.56E-01
PU-H71	HSP90	30	-0.08	6.74E-01
XL888	HSP90	29	-0.11	5.56E-01
Dinaciclib	CDK1/2/5/9	50	-0.12	4.12E-01
LN 2314	EGFR	33	-0.13	4.54E-01
UNC2250	Mer	28	-0.13	4.96E-01
LN2084/MJ 134.1c	EGFR	13	-0.14	6.43E-01
LN 2149/GM 373	EGFR	32	-0.15	4.24E-01
LN 2381/MJ 410	EGFR	32	-0.15	4.28E-01
LN 2313	EGFR	14	-0.15	6.06E-01
LN 2315	EGFR	32	-0.16	3.69E-01
Osimertinib	EGFR	53	-0.17	2.36E-01
LN 1994/GM-154	EGFR	33	-0.17	3.52E-01
Cediranib free base	VEGFR2	30	-0.17	3.70E-01
LN2009/MJ-120-2	EGFR	14	-0.17	5.52E-01
LN 2298	EGFR	33	-0.18	3.11E-01
Vandetanib	VEGFR2/3, EGFR	26	-0.18	3.73E-01
LN2095/MJ 195-197.1	EGFR	13	-0.24	4.37E-01
LN 2251/DNA19	EGFR	13	-0.25	4.15E-01

LN 2058/GM-301	EGFR	12	-0.29	3.66E-01
LN2124/MJ 080-2	EGFR	13	-0.38	1.96E-01

GDSC1 (b)

**Compound**	**Target**	**CLs (*n*=)**	**ρ (Spearman test)**	**P-value**
Nutlin-3a (-)	MDM2	127	0.62	1.10E-14
Imatinib	ABL, KIT, PDGFR	55	0.42	1.54E-03
Cyclopamine	SMO	54	0.39	3.18E-03
KIN001-135	IKKE	55	0.39	3.40E-03
Methotrexate	Dihydrofolate reductase (DHFR)	129	0.37	1.65E-05
PD-173074	FGFR1, FGFR3	127	0.37	2.37E-05
SL 0101-1	RSK, AURKB, PIM3	122	0.36	5.75E-05
Veliparib	PARP1, PARP2	129	0.33	1.25E-04
PF-4708671	p70 S6KA	140	0.32	9.90E-05
UNC1215	L3MBTL3	141	0.32	1.02E-04
XMD14-99	EPHB3, CAMK1	142	0.32	1.19E-04
ATRA	Retinoic acid and retinoid X receptor agonist	127	0.32	2.06E-04
VX-702	p38	128	0.32	2.66E-04
AZ628	BRAF	54	0.32	1.75E-02
MS-275	HDAC	54	0.32	1.80E-02
S-Trityl-L-cysteine	KIF11	53	0.31	2.19E-02
Salubrinal	GADD34-PP1C phosphatase	54	0.31	2.49E-02
Crizotinib	MET, ALK	55	0.3	2.49E-02
TGX221	PI3K beta	55	0.3	2.77E-02
STF-62247	Autophagy	142	0.29	4.78E-04
FK866	NAMPT inhibitor	138	0.29	6.85E-04
GNF-2	ABL (T315I)	53	0.29	3.64E-02
Tivozanib	VEGFR	142	0.28	6.47E-04
Dabrafenib	BRAF	132	0.28	1.28E-03
Nilotinib	ABL	124	0.28	1.45E-03
CEP-701	FLT3, JAK2, NTRK1, RET	128	0.28	1.46E-03
SGC0946	DOT1L (Q8TEK3)	140	0.27	1.40E-03
SB590885	BRAF	124	0.27	2.30E-03
PHA-665752	MET	55	0.27	4.57E-02
TL-2-105	CRAF	142	0.26	1.51E-03
AZD8055	mTORC1/2	126	0.26	2.91E-03
CGP-082996	CDK4	53	0.26	6.07E-02
AP-24534	ABL	143	0.25	2.33E-03
AC220	FLT3	142	0.25	2.47E-03
T0901317	LXR	142	0.25	2.76E-03
NU-7441	DNAPK	127	0.25	5.00E-03
AG-014699	PARP1, PARP2	142	0.24	3.37E-03
OSI-930	KIT, VEGFR, PDGFR	142	0.24	3.65E-03
EHT 1864	Rac GTPases	143	0.24	3.91E-03
Olaparib	PARP1, PARP2	267	0.23	1.95E-04
GW-2580	cFMS	142	0.23	5.68E-03

IPA-3	PAK	143	0.23	6.61E-03
(5Z)-7-Oxozeaenol	TAK1 (MAP3K7)	142	0.23	6.67E-03
Lenalidomide	TNF alpha	129	0.23	7.38E-03
Vorinostat	HDAC inhibitor Class I, IIa, IIb, IV	129	0.23	9.18E-03
681640	WEE1, CHEK1	117	0.23	1.36E-02
Z-LLNle-CHO	gamma-secretase	53	0.23	9.75E-02
MP470	PDGFR	142	0.22	7.91E-03
Ruxolitinib	JAK1, JAK2, TYK2	142	0.22	7.95E-03
TL-1-85	TAK	142	0.22	9.76E-03
GDC0449	SMO	129	0.22	1.19E-02
Camptothecin	TOP1	129	0.22	1.38E-02
ZM-447439	AURKB	120	0.22	1.49E-02
MLN4924	NEDD8-activating enzyme (NAE)	115	0.22	1.87E-02
SB 216763	GSK3A, GSK3B	112	0.22	2.23E-02
Sunitinib	PDGFRA, PDGFRB, KDR, KIT, FLT3	53	0.22	1.09E-01
BHG712	EPHB4	142	0.21	1.21E-02
Linifanib	RTK	142	0.21	1.42E-02
KU-55933	ATM	126	0.21	1.73E-02
BMS-708163	gamma-secretase	285	0.2	6.25E-04
KIN001-236	TIE2	142	0.2	1.55E-02
XMD15-27	CAMK2B, CLK2, DYRK1A, MAST1, STK39	142	0.2	1.60E-02
BAY 61-3606	SYK	141	0.2	1.80E-02
SN-38	TOP1	145	0.2	1.82E-02
XMD11-85h	BRSK2, FLT4, MARK4, PRKCD, RET, SPRK1	66	0.2	1.12E-01
Roscovitine	CDK family	53	0.2	1.62E-01
YM201636	PYKfyve (FYV1)	142	0.19	2.10E-02
XL-184	VEGFR, MET, RET, KIT, FLT1, FLT3, FLT4, TIE2, AXL	142	0.19	2.21E-02
PAC-1	CASP3 agonist	142	0.19	2.24E-02
EX-527	SIRT1	142	0.19	2.66E-02
Talazoparib	PARP1, PARP2	140	0.19	2.85E-02
CHIR-99021	GSK3B	284	0.18	2.90E-03
GSK1904529A	IGF1R	143	0.18	2.74E-02
FR-180204	ERK	142	0.18	3.32E-02
BIX02189	MEK5	142	0.18	3.39E-02
Pazopanib	VEGFR, PDGFRA, PDGFRB, KIT	142	0.18	3.55E-02
rTRAIL	DR4, DR5	143	0.18	3.60E-02
Navitoclax	BCL-2, BCL-XL, BCL-W	127	0.18	3.88E-02
MG-132	Proteasome	55	0.18	1.81E-01
NSC-87877	SHP-1 (PTPN6), SHP-2 (PTPN11)	143	0.17	3.84E-02
QS11	ARFGAP	143	0.17	4.78E-02
AICAR	AMPK agonist	127	0.17	5.25E-02
PD-0332991	CDK4, CDK6	122	0.17	6.48E-02
PLX4720	BRAF	269	0.16	6.96E-03
GSK269962A	ROCK1, ROCK2	199	0.16	2.38E-02
IOX2	EGLN1	144	0.16	4.83E-02
KIN001-270	CDK9	142	0.16	5.44E-02
Doxorubicin	DNA intercalating	143	0.16	5.76E-02
CH5424802	ALK	141	0.16	5.91E-02

PFI-1	BRD2, BRD3, BRD4	145	0.16	5.95E-02
Tubastatin A	HDAC6	142	0.16	6.19E-02
Rapamycin	mTOR	50	0.16	2.55E-01
XMD8-85	ERK5 (MK07)	54	0.16	2.58E-01
JQ1	BRD2, BRD3, BRD4	428	0.15	2.02E-03
BMS-536924	IGF1R	197	0.15	3.10E-02
NG-25	TAK	142	0.15	6.61E-02
CCT007093	PPM1D	143	0.15	6.71E-02
I-BET-762	BRD2, BRD3, BRD4	142	0.15	6.82E-02
Mitomycin C	DNA crosslinker	143	0.15	6.83E-02
FMK	RSK	134	0.15	7.48E-02
KIN001-244	PDK1 (PDPK1)	142	0.15	7.62E-02
Phenformin	NA	140	0.15	7.65E-02
JQ12	HDAC	143	0.15	8.37E-02
MK-2206	AKT1, AKT2	122	0.15	9.68E-02
TAE684	ALK	55	0.15	2.76E-01
Sorafenib	PDGFRA, PDGFRB, KDR, KIT, FLT3	55	0.15	2.78E-01
VX-11e	ERK	142	0.14	8.52E-02
SB 505124	TGFbetaR-I (ALK5)	145	0.14	8.61E-02
FTI-277	Farnesyl-transferase (FNTA)	143	0.14	8.76E-02
Etoposide	TOP2	143	0.14	9.16E-02
5-Fluorouracil	DNA antimetabolite	142	0.14	9.26E-02
TAK-715	p38a	142	0.14	9.41E-02
KIN001-266	TPL2,COT(M3K8)	142	0.14	9.72E-02
VNLG/124	HDAC,RAR	141	0.14	9.88E-02
Temozolomide	DNA akylating agent	141	0.14	1.04E-01
MPS-1-IN-1	MPS1	142	0.13	1.10E-01
PHA-793887	CDK-pan	142	0.13	1.12E-01
AR-42	HDAC	141	0.13	1.14E-01
Y-39983	ROCK	142	0.13	1.14E-01
Zibotentan	Endothelin A Receptor	142	0.13	1.16E-01
TG101348	JAK2	142	0.13	1.23E-01
KIN001-102	AKT1	141	0.13	1.25E-01
BX-912	PDK1 (PDPK1)	141	0.13	1.25E-01
GSK1070916	AURKB	135	0.13	1.32E-01
KIN001-260	IKK	142	0.13	1.35E-01
Cisplatin	DNA crosslinker	129	0.13	1.44E-01
selumetinib	MEK1, MEK2	265	0.12	4.69E-02
Midostaurin	KIT	143	0.12	1.41E-01
ZSTK474	PI3K	141	0.12	1.45E-01
AS605240	PI3K gamma	142	0.12	1.48E-01
Belinostat	HDAC	138	0.12	1.52E-01
Bleomycin (50 uM)	DNA damage	145	0.12	1.53E-01
LAQ824	HDAC	143	0.12	1.56E-01
CAY10603	HDAC6	141	0.12	1.58E-01
JNJ-26854165	MDM2	142	0.12	1.62E-01
LFM-A13	BTK	143	0.12	1.62E-01
Axitinib	PDGFR, KIT, VEGFR	128	0.12	1.81E-01

BEZ235	PI3K (class 1), mTORC1/2	126	0.12	1.90E-01
QL-VIII-58	mTOR, ATR	66	0.12	3.43E-01
UNC0638	G9a(EHMT2), GLP(EHMT1)	286	0.11	7.22E-02
Tamoxifen	ER	145	0.11	1.72E-01
Masitinib	KIT	142	0.11	1.75E-01
NPK76-II-72-1	PLK3	142	0.11	1.83E-01
OSI-027	mTORC1/2	142	0.11	1.85E-01
CP466722	ATM	141	0.11	1.88E-01
WZ3105	CLK2, CNSK1E, FLT3, ULK1	142	0.11	1.95E-01
QL-XI-92	DDR1	142	0.11	2.02E-01
HG-6-64-1	BRAFV600E, TAK, MAP4K5	142	0.11	2.06E-01
Lisitinib	IGF1R	143	0.11	2.10E-01
JNK Inhibitor VIII	JNK	127	0.11	2.13E-01
BX-795	TBK1, PDPK1, IKK, AURKB, AURKC	127	0.11	2.23E-01
GW843682X	PLK1	53	0.11	4.21E-01
GDC0941	PI3K	265	0.1	9.66E-02
Bleomycin	DNA damage	285	0.1	1.00E-01
GSK429286A	ROCK2	141	0.1	2.18E-01
JNK-9L	JNK, CDK9	143	0.1	2.20E-01
XMD13-2	RIPK	142	0.1	2.22E-01
JW-7-24-1	LCK	142	0.1	2.33E-01
CAL-101	PI3K delta	142	0.1	2.33E-01
Shikonin	unknown	143	0.1	2.34E-01
TPCA-1	IKK	142	0.1	2.44E-01
GSK-650394	SGK3	140	0.1	2.56E-01
CCT018159	HSP90	135	0.1	2.56E-01
PIK-93	PI4K, PI3K	142	0.1	2.60E-01
SNX-2112	HSP90	141	0.1	2.60E-01
Bicalutamide	Androgen receptor (ANDR)	279	0.09	1.34E-01
CX-5461	RNA Pol I	142	0.09	2.75E-01
AZD7762	CHEK1, CHEK2	129	0.09	2.87E-01
NSC-207895	MDMX	142	0.09	3.04E-01
CI-1040	MEK1, MEK2	126	0.09	3.08E-01
piperlongumine	Increases ROS levels	145	0.09	3.08E-01
BMS345541	IKBKB	142	0.09	3.12E-01
Foretinib	MET	140	0.09	3.16E-01
QL-XII-61	BMX, BTK	62	0.09	4.66E-01
Gemcitabine	DNA replication	143	0.08	3.17E-01
Genentech Cpd 10	AURKA, AURKB	142	0.08	3.20E-01
Cetuximab	EGFR	138	0.08	3.22E-01
Bryostatin 1	PRKC	143	0.08	3.24E-01
OSU-03012	PDK1 (PDPK1)	143	0.08	3.54E-01
AMG-706	VEGFR, RET, c-KIT, PDGFR	127	0.08	3.59E-01
THZ-2-102-1	CDK7	140	0.08	3.62E-01
Trametinib	MEK1, MEK2	136	0.08	3.81E-01
RO-3306	CDK1	127	0.08	3.90E-01
HG-5-88-01	EGFR, ADCK4	66	0.08	5.04E-01
Parthenolide	NFKB1	55	0.08	5.70E-01

AZD6482	PI3K beta (P3C2B)	284	0.07	2.15E-01
THZ-2-49	CDK9	141	0.07	3.85E-01
TW 37	BCL-2, BCL-XL	142	0.07	4.00E-01
Cytarabine	DNA synthesis	129	0.07	4.01E-01
AT-7519	CDK9	141	0.07	4.05E-01
CUDC-101	HDAC, EGFR	140	0.07	4.43E-01
VX-680	AURKA, AURKB, AURKC, FLT3, ABL1, JAK2	52	0.07	6.08E-01
PI-103	PI3K alpha, DNAPK	141	0.06	4.64E-01
XAV939	TNKS1, TNKS2	143	0.06	4.66E-01
CP724714	ERBB2	142	0.06	5.02E-01
HG-5-113-01	LOK, LTK, TRCB, ABL(T315I)	66	0.06	6.49E-01
CGP-60474	CDK1, CDK2, CDK5, CDK7, CDK9	53	0.06	6.53E-01
Paclitaxel	Beta subunit of Tubulin	53	0.06	6.68E-01
RDEA119	MEK1, MEK2	264	0.05	4.61E-01
GSK690693	AKT	141	0.05	5.22E-01
AKT inhibitor VIII	AKT1, AKT2, AKT3	143	0.05	5.30E-01
AS601245	JNK	143	0.05	5.52E-01
A-443654	AKT1, AKT2, AKT3	53	0.05	7.38E-01
Ispinesib Mesylate	KIF11	141	0.04	6.24E-01
GSK2126458	PI3K, mTOR	142	0.04	6.68E-01
GW 441756	NTRK1	129	0.04	6.72E-01
Erlotinib	EGFR	49	0.04	7.70E-01
Embelin	XIAP	143	0.03	6.87E-01
YK 4-279	RNA helicase A	128	0.03	7.13E-01
Vinblastine	Microtubules	129	0.03	7.19E-01
Temsirolimus	mTOR	128	0.03	7.23E-01
QL-X-138	MNK2, DNAPK, MTOR, BTK, JAK3	139	0.03	7.32E-01
Pyrimethamine	Dihydrofolate reductase (DHFR)	55	0.03	8.14E-01
Saracatinib	SRC, ABL1	55	0.03	8.32E-01
Bexarotene	Retinioic acid X family agonist	141	0.02	7.72E-01
BMS-754807	IGF1R	142	0.02	7.74E-01
Obatoclax Mesylate	BCL-2, BCL-XL, MCL-1	143	0.02	7.91E-01
SB52334	ALK5	142	0.02	8.24E-01
Bosutinib	SRC, ABL, TEC	129	0.02	8.37E-01
BMS-509744	ITK	53	0.02	8.72E-01
WH-4-023	SRC family, ABL	53	0.02	8.96E-01
Tipifarnib	Farnesyl-transferase (FNTA)	143	0.01	9.06E-01
KIN001-055	JAK3, MNK1	142	0.01	9.16E-01
Vinorelbine	Microtubules	143	0.01	9.27E-01
Bortezomib	Proteasome	54	0.01	9.36E-01
FH535	unknown	143	0	9.59E-01
AUY922	HSP90	141	0	9.63E-01
LY317615	PKCbeta	141	0	9.63E-01
BI-2536	PLK1, PLK2, PLK3	53	0	9.78E-01
QL-XII-47	BTK, BMX	140	0	9.87E-01
Gefitinib	EGFR	127	0	9.93E-01
Dasatinib	ABL, SRC, KIT, PDGFR	53	0	9.93E-01
DMOG	Prolyl-4-Hydroxylase	143	-0.01	8.64E-01

XMD8-92	ERK5	66	-0.01	9.24E-01
JW-7-52-1	mTOR	52	-0.01	9.38E-01
EKB-569	EGFR	142	-0.02	7.79E-01
PF-562271	FAK	140	-0.03	7.26E-01
BIRB 0796	Androgen receptor (ANDR)	127	-0.03	7.29E-01
PD-0325901	MEK1, MEK2	126	-0.04	6.76E-01
ZG-10	IRAK1	66	-0.05	6.94E-01
Epothilone B	Microtubules	143	-0.06	4.50E-01
A-770041	SRC family	53	-0.09	5.28E-01
Thapsigargin	ATPase, Ca++ transporting, cardiac muscle, slow twitch 2	143	-0.11	2.00E-01
Afatinib	ERBB2, EGFR	270	-0.12	4.09E-02
17-AAG	HSP90	127	-0.12	1.63E-01
Lapatinib	EGFR, ERBB2	53	-0.15	2.69E-01
WZ-1-84	BMX	53	-0.16	2.42E-01
YM155	BIRC5 (Survivin)	138	-0.17	4.88E-02
Elesclomol	HSP70	129	-0.17	4.88E-02
CMK	RSK	53	-0.17	2.28E-01
Docetaxel	Microtubules	129	-0.2	2.43E-02

GDSC2 (c)

**Compound**	**Target**	**CLs (*n*=)**	**ρ (Spearman test)**	**P-value**
Nutlin-3a (-)	MDM2	132	0.61	<2.2E-16
SB216763	GSK3A, GSK3B	22	0.47	3.02E-02
JQ1	BRD2, BRD3, BRD4, BRDT	12	0.44	1.54E-01
Doramapimod	p38, JNK2	12	0.42	1.77E-01
PD173074	FGFR1, FGFR2, FGFR3	130	0.42	1.13E-06
I-BRD9	BRD9	130	0.38	8.08E-06
Oxaliplatin	DNA alkylating agent	135	0.38	5.62E-06
Ruxolitinib	JAK1, JAK2	129	0.38	1.37E-05
BMS-345541	IKK-1, IKK-2	129	0.37	1.64E-05
Vorinostat	HDAC inhibitor Class I, IIa, IIb, IV	130	0.37	2.22E-05
tozasertib	AURKA, AURKB, AURKC, others	12	0.36	2.46E-01
ERK_6604	ERK1, ERK2	129	0.36	3.78E-05
MK-8776	CHEK1, CHEK2, CDK2	130	0.36	3.56E-05
Ribociclib	CDK4, CDK6	11	0.35	2.86E-01
Fulvestrant	ESR	135	0.35	3.18E-05
Telomerase Inhibitor IX	Telomerase	130	0.35	4.90E-05
MIRA-1	TP53	130	0.35	5.94E-05
Dabrafenib	BRAF	131	0.35	5.90E-05
Pevonedistat	NAE	130	0.34	7.32E-05
Wnt-C59	PORCN	128	0.34	1.11E-04
AZD2014	mTORC1, mTORC2	129	0.34	1.10E-04
Palbociclib	CDK4, CDK6	131	0.33	1.26E-04
Epirubicin	Anthracycline	130	0.33	1.38E-04
AGI-6780	IDH2 R140Q mutant	128	0.32	2.01E-04
AZD5153	BRD4	129	0.32	2.10E-04
AMG-319	PI3K (beta sparing)	131	0.32	1.88E-04

ERK_2440	ERK1, ERK2	129	0.32	2.43E-04
Sabutoclax	BCL2, BCL-XL, BFL1, MCL1	127	0.32	2.74E-04
JAK1_8709	JAK1	129	0.32	2.73E-04
Leflunomide	Pyrimidine synthesis inhibitor	129	0.32	2.77E-04
BDP-00009066	MRCKB_HUMAN	130	0.32	2.77E-04
Buparlisib	PI3Kalpha, PI3Kdelta, PI3Kbeta, PI3Kgamma	130	0.31	3.12E-04
IWP-2	PORCN	129	0.31	3.46E-04
Selumetinib	MEK1, MEK2	129	0.31	3.98E-04
Linsitinib	IGF1R	135	0.31	2.92E-04
AZD4547	FGFR1, FGFR2, FGFR3	130	0.31	3.89E-04
CDK9_5038	CDK9	129	0.31	4.30E-04
Sorafenib	PDGFR, KIT, VEGFR, RAF	130	0.3	4.65E-04
AGI-5198	IDH1 (R132H)	130	0.3	5.19E-04
Venetoclax	BCL2	130	0.3	5.34E-04
Ulixertinib	ERK1, ERK2	132	0.3	4.89E-04
MK-2206	AKT1, AKT2	132	0.3	4.95E-04
Luminespib	HSP90	135	0.3	4.32E-04
VSP34_8731	VSP34	129	0.3	5.87E-04
Savolitinib	MET	130	0.3	5.90E-04
CDK9_5576	CDK9	129	0.3	6.84E-04
Wee1 Inhibitor	WEE1, CHEK1	130	0.3	6.78E-04
AZD1208	PIM1, PIM2, PIM3	129	0.29	7.26E-04
Mirin	MRE11	129	0.29	8.33E-04
5-Fluorouracil	Antimetabolite (DNA & RNA)	135	0.29	6.35E-04
Camptothecin	TOP1	135	0.29	6.58E-04
BPD-00008900		130	0.29	8.57E-04
Carmustine	Alkylating agent	128	0.29	9.47E-04
LGK974	PORCN	135	0.29	7.57E-04
OF-1	BRPF1B, BRPF2	128	0.29	1.06E-03
Cytarabine	Antimetabolite	130	0.29	9.89E-04
Irinotecan	TOP1	135	0.29	8.20E-04
AZD5438	CDK2	129	0.28	1.12E-03
Niraparib	PARP1, PARP2	129	0.28	1.15E-03
Cediranib	VEGFR, FLT1, FLT2, FLT3, FLT4, KIT, PDGFRB	130	0.28	1.12E-03
ML323	USP1, UAF1	128	0.28	1.23E-03
VX-11e	ERK2	128	0.28	1.29E-03
Cyclophosphamide	Alkylating agent	130	0.28	1.35E-03
EPZ004777	DOT1L	135	0.27	1.40E-03
Gemcitabine	Pyrimidine antimetabolite	131	0.27	1.67E-03
Axitinib	PDGFR, KIT, VEGFR	12	0.27	3.91E-01
IAP_5620	IAP	127	0.27	2.05E-03
Foretinib	MET, KDR, TIE2, VEGFR3/FLT4, RON, PDGFR, FGFR1, EGFR	131	0.27	1.78E-03
NVP-ADW742	IGF1R	130	0.27	1.90E-03
Elephantin		128	0.27	2.11E-03
BIBR-1532	TERT	131	0.27	1.93E-03
LJI308	RSK2, RSK1, RSK3	128	0.27	2.19E-03
AZ6102	TNKS1, TNKS2	128	0.27	2.19E-03
VE821	ATR	128	0.27	2.26E-03

Nilotinib	ABL	130	0.27	2.18E-03
BI-2536	PLK1, PLK2, PLK3	11	0.26	4.35E-01
Crizotinib	MET, ALK, ROS1	129	0.26	3.43E-03
Entinostat	HDAC1, HDAC3	128	0.26	3.59E-03
TAF1_5496	TAF1	129	0.26	3.58E-03
Zoledronate		128	0.25	4.01E-03
AZD5363	AKT1, AKT2, AKT3, ROCK2	130	0.25	3.86E-03
Nelarabine		128	0.25	4.20E-03
Temozolomide	DNA alkylating agent	130	0.25	4.48E-03
Cisplatin	DNA crosslinker	132	0.25	4.36E-03
EPZ5676	DOT1L	135	0.25	4.17E-03
Olaparib	PARP1, PARP2	130	0.24	5.25E-03
PAK_5339	PAK1, PAK2	129	0.24	5.74E-03
Dactinomycin	RNA polymerase	135	0.24	4.85E-03
PFI3	Polybromo 1, SMARCA4, SMARCA2	128	0.24	6.20E-03
Vinorelbine	Microtubule destabiliser	128	0.24	6.77E-03
Tamoxifen	ESR1	130	0.24	6.46E-03
Dactolisib	PI3K (class 1), MTORC1, MTORC2	130	0.24	6.60E-03
ABT737	BCL2, BCL-XL, BCL-W, BCL-B, BFL1	129	0.24	7.29E-03
PRIMA-1MET	TP53 activation	129	0.23	7.66E-03
Afuresertib	AKT1, AKT2, AKT3	130	0.23	7.44E-03
GSK2578215A	LRRK2	130	0.23	7.61E-03
CZC24832	PI3Kgamma	128	0.23	8.20E-03
I-BET-762	BRD2, BRD3, BRD4	128	0.23	8.39E-03
AZD5991	MCL1	129	0.23	8.46E-03
Gallibiscoquinazole		128	0.23	9.37E-03
PD0325901	MEK1, MEK2	135	0.23	7.62E-03
SCH772984	ERK1, ERK2	134	0.23	7.86E-03
Bortezomib	Proteasome	130	0.23	8.89E-03
AZD1332	NTRK1, NTRK2, NTRK3	129	0.23	9.25E-03
AZD8055	MTORC1, MTORC2	22	0.23	3.05E-01
MIM1	MCL1	130	0.23	9.01E-03
Daporinad	NAMPT	59	0.23	8.16E-02
OTX015	BRD2, BRD3, BRD4	128	0.23	9.75E-03
GSK343	EZH2	128	0.23	9.81E-03
Picolinici-acid	Inflammatory related	128	0.23	1.03E-02
PRT062607	SYK	128	0.23	1.08E-02
JAK_8517	JAK1, JAK2	129	0.22	1.09E-02
Teniposide	TOP2	128	0.22	1.18E-02
AZD6738	ATR	130	0.22	1.14E-02
Eg5_9814	KSP11	127	0.22	1.28E-02
Mitoxantrone	TOP2, PKC	128	0.22	1.46E-02
Vinblastine	Microtubule destabiliser	130	0.22	1.39E-02
BMS-536924	IGF1R, IR	130	0.22	1.41E-02
RVX-208	BRD4	128	0.21	1.50E-02
GSK1904529A	IGF1R, IR	135	0.21	1.27E-02
Alpelisib	PI3Kalpha	135	0.21	1.28E-02
Pictilisib	PI3K (class 1)	131	0.21	1.47E-02

Obatoclax Mesylate	BCL2, BCL-XL, BCL-W, MCL1	129	0.21	1.63E-02
Talazoparib	PARP1, PARP2	128	0.21	1.76E-02
GSK2606414	PERK	127	0.21	1.81E-02
AZD7762	CHEK1, CHEK2	130	0.21	1.73E-02
AT13148	AKT1	128	0.21	1.92E-02
IGF1R_3801	IGFR1	129	0.21	1.88E-02
PCI-34051	HDAC8, HDAC6, HDAC1	128	0.2	2.05E-02
Ipatasertib	AKT1, AKT, AKT3	130	0.2	2.00E-02
Navitoclax	BCL2, BCL-XL, BCL-W	127	0.2	2.15E-02
GNE-317	PI3Kalpha	128	0.2	2.26E-02
Uprosertib	AKT1, AKT2, AKT3	133	0.2	2.05E-02
Dinaciclib	CDK1, CDK2, CDK5, CDK9	128	0.2	2.63E-02
ULK1_4989	ULK1	129	0.2	2.64E-02
WZ4003	NUAK1, NUAK2	128	0.19	2.91E-02
AZD8186	PI3Kalpha, PI3Kbeta	130	0.19	2.80E-02
Fludarabine	STAT1	128	0.19	3.02E-02
Trametinib	MEK1, MEK2	132	0.19	2.78E-02
GDC0810	ESR1, ESR2	130	0.19	3.08E-02
AZ960	JAK2, JAK3	129	0.19	3.28E-02
Rapamycin	MTORC1	128	0.18	3.85E-02
GSK591	PMRT5	128	0.18	4.09E-02
Staurosporine	Broad spectrum kinase inhibitor	132	0.18	3.80E-02
MG-132	Proteasome, CAPN1	132	0.18	4.10E-02
Vincristine	Microtubule destabiliser	126	0.17	5.17E-02
LY2109761	TGFB1	128	0.17	5.23E-02
IRAK4_4710	IRAK4	129	0.17	5.50E-02
P22077	USP7, USP47	130	0.17	5.52E-02
NU7441	DNAPK	12	0.17	6.04E-01
VE-822	ATR	128	0.17	5.98E-02
YK-4-279	RNA helicase A	130	0.17	5.94E-02
Podophyllotoxin bromide		128	0.17	6.22E-02
PLX-4720	BRAF	134	0.16	5.96E-02
RO-3306	CDK1	12	0.16	6.19E-01
XAV939	TNKS1, TNKS2	129	0.16	7.01E-02
Sinularin		128	0.16	7.43E-02
Dihydrorotenone		128	0.16	7.72E-02
Alisertib	AURKA	129	0.15	8.35E-02
KRAS (G12C) Inhibitor-12	KRAS (G12C)	128	0.15	9.10E-02
Pyridostatin	G-quadruplex stabiliser	131	0.14	1.01E-01
Topotecan	TOP1	128	0.14	1.18E-01
Paclitaxel	Microtubule stabiliser	130	0.14	1.17E-01
MK-1775	WEE1, PLK1	132	0.13	1.35E-01
LCL161	XIAP, IAP1, IAP2	129	0.11	1.95E-01
UMI-77	MCL1	130	0.1	2.50E-01
AZD6482	PI3Kbeta	12	0.1	7.66E-01
Docetaxel	Microtubule stabiliser	134	0.1	2.65E-01
Taselisib	PI3K (beta sparing)	135	0.09	2.98E-01

MN-64	TNKS1, TNKS2	128	0.08	3.86E-01
Entospletinib	SYK	128	0.08	3.98E-01
WIKI4	TNKS1, TNKS2	130	0.07	4.51E-01
WEHI-539	BCL-XL	130	0.06	5.22E-01
OSI-027	MTORC1, MTORC2	47	0.05	7.19E-01
Osimertinib	EGFR	130	0.04	6.30E-01
AZD5582	XIAP, cIAP	128	0.04	6.62E-01
AZD3759	EGFR	131	0.03	7.19E-01
Gefitinib	EGFR	130	0.03	7.21E-01
Acetalax		128	0.03	7.73E-01
PF-4708671	S6K1	12	0.01	9.74E-01
SB505124	TGFBR1, ACVR1B, ACVR1C	10	-0.01	1.00E+00
KU-55933	ATM	12	-0.01	9.91E-01
Erlotinib	EGFR	130	-0.02	8.56E-01
Sepantronium bromide	BIRC5	130	-0.02	8.48E-01
Lapatinib	EGFR, ERBB2	130	-0.04	6.73E-01
Afatinib	ERBB2, EGFR	135	-0.07	4.31E-01
Ibrutinib	BTK	128	-0.09	3.22E-01
Sapitinib	EGFR, ERBB2, ERBB3	135	-0.13	1.26E-01
Dasatinib	ABL, SRC, Ephrins, PDGFR, KIT	131	-0.17	4.92E-02
GSK269962A	ROCK1, ROCK2	12	-0.2	5.28E-01
ZM447439	AURKA, AURKB	12	-0.24	4.57E-01
BMS-754807	IGF1R, IR	10	-0.33	3.49E-01

Supplementary Table 4. Distribution of the molecularly annotated 237 CLs across tumour (sub)types and MI-773 drug response classes.

	Highly sensitive (Hs) < 1 µM	Intermediate sensitive (Is) [1, 10[µM	Resistant ≥ 10 µM	Total	% Sensitive (Hs +Is)
**CLs from solid tumours**					
Melanoma	5	2	3	10	70
Kidney	4	2	6	12	50
Mesothelioma	2	0	2	4	50
Sarcoma	2	2	6	10	40
Liver	1	3	7	11	36
CNS	1	2	6	9	33
Stomach	3	2	10	15	33
Bladder	0	2	5	7	29
CRC	1	3	11	15	27
Prostate	0	1	3	4	25
Breast	1	2	11	14	21
Ovary	0	2	8	10	20
HNSC	2	0	10	12	17
NSCLC	0	4	21	25	16
SCLC	1	0	9	10	10
Miscellaneous	0	0	1	1	0
Oesophagus	0	0	5	5	0
Pancreas	0	0	15	15	0
Uterus	0	0	8	8	0
**Total**	**23**	**27**	**147**	**197**	
**CLs from haematological cancers**					
LY_Hodgkin	0	1	0	1	100
LY_unclass	0	2	1	3	67
LY_MM	3	2	4	9	56
LY_Burkitt	1	0	1	2	50
LE_ALL	2	0	2	4	50
LE_AML	4	1	5	10	50
LE_CLL	0	1	0	1	100
LE_CML	0	0	6	6	0
LY_DLBC	0	0	4	4	0
**Total**	**10**	**7**	**23**	**40**	
**Total**	**33**	**34**	**170**	**237**	

Supplementary Table 5. MI-773 drug response classes and *TP53* mutational status.

	**Sensitive (< 10 µM)**	**Resistant (≥ 10 µM)**	**Total**
wild type *TP53*	53	21	74
mutated *TP53*	14	149	163
P-value (Fisher test)	<2.2E-16		
**Total**	67	170	237

Supplementary Table 6: Pathway enrichment analysis using gene transcripts positively associated with MI-773 response.

(a) Kyoto Encyclopedia of Genes and Genomes (KEGG)

Description	GeneRatio	BgRatio	p-value	p-adjust	q-value	Gene ID	Gene Count
p53 signalling pathway	[11/52]	72/8039	6.08E-13	9.30E-11	7.87E-11	BCL2 / CDKN1A / ZMAT3 / DDB2 / MDM2 / BAX / RRM2B / SESN1 / MDM4 / PPM1D / CCNG1	11
Glioma	[5/52]	75/8039	0.000114	0.005817	0.004923	CDKN1A / CAMK2D / DDB2 / MDM2 / BAX	5
Thyroid cancer	[4/52]	37/8039	8.78E-05	0.005817	0.004923	CDKN1A / DDB2 / BAX / TPR	4
Transcriptional misregulation in cancer	[6/52]	186/8039	0.00119	0.030961	0.026201	CDKN1A / MEF2C / BCL2A1 / DDB2 / MDM2 / BAX	6
Melanoma	[4/52]	72/8039	0.001158	0.030961	0.026201	CDKN1A / DDB2 / MDM2 / BAX	4
Platinum drug resistance	[4/52]	73/8039	0.001219	0.030961	0.026201	BCL2 / CDKN1A / MDM2 / BAX	4
Chronic myeloid leukaemia	[4/52]	76/8039	0.001417	0.030961	0.026201	CDKN1A / DDB2 / MDM2 / BAX	4
Colorectal cancer	[4/52]	86/8039	0.002236	0.04276	0.036185	BCL2 / CDKN1A / DDB2 / BAX	4
Small cell lung cancer	[4/52]	92/8039	0.002859	0.048608	0.041134	BCL2 / CDKN1A / DDB2 / BAX	4

The BgRatio (background ratio) is the ratio of total genes in each subset to the whole database.
N: total genes in database (e.g., KEGG)
M: size of gene set (e.g., p53 pathway)
BgRatio = M/N
The GeneRatio: is the ratio of input genes (upregulated genes) found in each subset to the whole database.
n: overlap between input genes and genes in N (in whole KEGG database)
k: overlap between input genes and genes in M (e.g.in p53 pathway set)
GeneRatio = k/n

(b) Gene Ontology (GO): Biological Process (BP)

Description	GeneRatio	BgRatio	p-value	p-adjust	q-value	Gene ID	Gene Count
negative regulation of mitotic cell cycle	13/89	305/17381	4.94E-09	1.08E-05	9.39E-06	BCL2/CDKN1A/BTG2/RPS27L/MDM2/BAX/TPR/MDM4/XPC/USP47/CRADD/TNKS/TRIM35	13
intrinsic apoptotic signalling pathway	12/89	292/17381	3.03E-08	2.38E-05	2.06E-05	BCL2/CDKN1A/BCL2A1/ZMAT3/RPS27L/MDM2/BAX/RRM2B/AEN/USP47/ARHGEF2/LRRK2	12
DNA damage checkpoint	9/89	155/17381	9.78E-08	2.38E-05	2.06E-05	CDKN1A/BTG2/RPS27L/MDM2/BAX/MDM4/XPC/INTS7/CRADD	9
mitotic DNA damage checkpoint	8/89	102/17381	5.08E-08	2.38E-05	2.06E-05	CDKN1A/BTG2/RPS27L/MDM2/BAX/MDM4/XPC/CRADD	8
mitotic DNA integrity checkpoint	8/89	109/17381	8.55E-08	2.38E-05	2.06E-05	CDKN1A/BTG2/RPS27L/MDM2/BAX/MDM4/XPC/CRADD	8
DNA integrity checkpoint	9/89	164/17381	1.59E-07	2.90E-05	2.51E-05	CDKN1A/BTG2/RPS27L/MDM2/BAX/MDM4/XPC/INTS7/CRADD	9
signal transduction by p53 class mediator	10/89	270/17381	1.20E-06	9.73E-05	8.43E-05	CDKN1A/ZMAT3/BTG2/RPS27L/MDM2/BAX/RRM2B/AEN/MDM4/CRADD	10
cellular response to abiotic stimulus	10/89	296/17381	2.74E-06	0.0001751	0.0001517	CDKN1A/DDB2/MDM2/BAX/ABCB4/XPC/USP47/INTS7/ARHGEF2/CRADD	10
cellular response to environmental stimulus	10/89	296/17381	2.74E-06	0.0001751	0.0001517	CDKN1A/DDB2/MDM2/BAX/ABCB4/XPC/USP47/INTS7/ARHGEF2/CRADD	10
regulation of apoptotic signalling pathway	8/89	383/17381	0.0007711	0.0252518	0.0218801	BCL2/MDM2/BAX/RRM2B/USP47/ARHGEF2/CRADD/LRRK2	8

Supplementary Table 7. Gene expression clusters and MI-773 drug response classes.

	Cluster 1		Cluster 2		Cluster 3	
Number of sensitive CLs (means Abs IC_{50})	40 (1.95 µM)	20^{Hs}	17 (2.7 µM)	10^{Hs}	10 (2.73 µM)	3^{Hs}
		20^{Is}		7^{Is}		7^{Is}
Number of resistant CLs (means Abs IC_{50})	89 (16.73 µM)		16 (13.3 µM)		65 (17.72 µM)	
Total	129		33		75	
Response Rate (RR) %	31		52		13	
P-value (Proportion test)	0.0002					

Hs: Highly sensitive < 1 µM; Is: Intermediate sensitive [1, 10[µM

Supplementary Table 8. Association between CLs predictive score classes and *TP53* status.

	Number of CLs		
	TP53 MUT	*TP53* WT	Total
High score (> 7.5)	48	53	101
Low score (≤ 7.5)	115	21	136
P-value (Fisher test)	1.378E-09		
**Total**	163	74	237

Supplementary Table 9. Association of the predictive score and MI-773 response classes stratified by *TP53* status.

(a)	Number of CLs		Total
	Sensitive (Abs IC_{50} < 10 μM)	Resistant (Abs $IC_{50} \geq$ 10 μM)	
High score (> 7.5)	59 (58%)	42	101
Low score (≤ 7.5)	8	128 (94%)	136
Total	67	170	237
P-value (Fisher)	<2.2E-16		

(b)	Number of CLs		Total
	Sensitive (Abs IC_{50} < 10 μM)	Resistant (Abs $IC_{50} \geq$ 10 μM)	
High score (> 7.5)	48 (91%)	5	53
Low score (≤ 7.5)	5	16	21
Total	53	21	74
P-value (Fisher test)	3.973E-08		

(c)	Number of CLs		Total
	Sensitive (Abs IC_{50} < 10 μM)	Resistant (Abs $IC_{50} \geq$ 10 μM)	
High score (> 7.5)	11	37	48
Low score (≤ 7.5)	3	112 (97%)	115
Total	14	149	163
P-value (Fisher test)	0.0001004		

a) overall CLs; b) CLs *TP53* WT c); CLs *TP53* mutated

Supplementary Table 10. Evaluation of *TP53* status, the predictive score, and both combined to predict MI-773 response.

Statistic	*TP53* status	Predictive Score	*TP53* status + Predictive Score
	Value (95 % CIs)		
Apparent prevalence	0.31 (0.25, 0.38)	0.43 (0.36,0.49)	0.72 (0.60, 0.81)
True prevalence	0.28 (0.23, 0.34)	0.28 (0.23,0.34)	0.72 (0.60, 0.81)
Sensitivity	0.79 (0.67, 0.88)	0.88 (0.78, 0.95)	0.91 (0.79, 0.97)
Specificity	0.88 (0.82, 0.92)	0.75 (0.68, 0.82)	0.76 (0.53, 0.92)
Positive predictive value	0.72 (0.60, 0.81)	0.58 (0.48, 0.68)	0.91 (0.79, 0.97)
Negative predictive value	0.91 (0.86, 0.95)	0.94 (0.89, 0.97)	0.76 (0.53, 0.92)
Positive likelihood ratio	6.40 (4.21, 9.74)	3.56 (2.70, 4.70)	3.80 (1.76, 8.22)
Negative likelihood ratio	0.24 (0.15, 0.38)	0.16 (0.08, 0.31)	0.12 (0.05, 0.29)
Diagnostic accuracy	0.85 (0.8, 0.89)	0.79 (0.73, 0.84)	0.86 (0.77, 0.93)

Supplementary Table 11. The performance of *TP53* status, the predictive score, and both combined to predict Nutlin-3a response (GDSC1 dataset).

Statistic	*TP53* status	Predictive Score	*TP53* status + Predictive Score
	Value (95 % CIs)		
Apparent prevalence	0.30 (0.26, 0.35)	0.47 (0.43, 0.52)	0.83 (0.75, 0.89)
True prevalence	0.30 (0.26, 0.34)	0.30 (0.26, 0.34)	0.67 (0.58, 0.75)
Sensitivity	0.67 (0.58, 0.75)	0.82 (0.74, 0.88)	0.95 (0.89, 0.99)
Specificity	0.86 (0.81, 0.89)	0.67 (0.62, 0.72)	0.43 (0.28, 0.59)
Positive predictive value	0.67 (0.58, 0.75)	0.51 (0.44, 0.58)	0.77 (0.68, 0.85)
Negative predictive value	0.86 (0.82, 0.90)	0.90 (0.85, 0.93)	0.83 (0.61, 0.95)
Positive likelihood ratio	4.70 (3.49, 6.34)	2.49 (2.08, 2.98)	1.68 (1.29, 2.18)
Negative likelihood ratio	0.38 (0.30, 0.49)	0.27 (0.19, 0.39)	0.11 (0.04, 0.29)
Diagnostic accuracy	0.80 (0.76, 0.84)	0.72 (0.67, 0.76)	0.78 (0.7, 0.85)

Supplementary Table 12. Levels of significance (*p*-values*) of *TP53* status, the predictive score, and both combined for association with Abs IC_{50} of various p53 related inhibitory drugs.

	TP53 status	Predictive Score >7.5 (Yes/No)	Predictive Score >7.5 (Yes/No) in WT *TP53* CLs	*TP53* WT + Predictive Score >7.5 (Yes/No)
Nutlin-3a (GDSC2)	< 2.2e-16	< 2.2e-16	3.60E-09	2.20E-16
RG-7112 (4HF BIOTEC)	8.71E-11	< 2.2e-16	9.99E-11	2.20E-16
YH239-EE (4HF BIOTEC)	0.005	0.02	0.13	0.01
PRIMA-1MET (GDSC2)	0.01	1.88E-10	0.04	0.001
MIRA-1 (GDSC2)	0.05	1.10E-11	0.06	0.01
JNJ-26854165 (GDSC2)	0.03	0.12	0.25	0.02
NSC-207895 (GDSC1)	0.43	5.00E-04	5.00E-04	0.11

**p*-value determined by Wilcoxon test.
