# Supplementary material for: Pharmacogenomics characterization of the MDM2 inhibitor MI-773 reveals candidate tumours and predictive biomarkers
Source: NPJ Precis Oncol. 2021 Oct 28;5:96. doi: 10.1038/s41698-021-00235-7 (PMC8553758; doi:10.1038/s41698-021-00235-7)
Supplement: Supplementary file 2 — Reporting Summary [file 41698_2021_235_MOESM2_ESM.pdf]

nature research

Corresponding author(s): Vincent Vuaroqueaux

Last updated by author(s): Jun 2, 2020

Reporting Summary

Nature Research wishes to improve the reproducibility of the work that we publish. This form provides structure for consistency and transparency in reporting. For further information on Nature Research policies, see our Editorial Policies and the Editorial Policy Checklist.

Statistics

For all statistical analyses, confirm that the following items are present in the figure legend, table legend, main text, or Methods section.

n/a	Confirmed	
☐	☒	The exact sample size (n) for each experimental group/condition, given as a discrete number and unit of measurement
☐	☒	A statement on whether measurements were taken from distinct samples or whether the same sample was measured repeatedly
☐	☒	The statistical test(s) used AND whether they are one- or two-sided<br>*Only common tests should be described solely by name; describe more complex techniques in the Methods section.*
☒	☐	A description of all covariates tested
☐	☒	A description of any assumptions or corrections, such as tests of normality and adjustment for multiple comparisons
☐	☒	A full description of the statistical parameters including central tendency (e.g. means) or other basic estimates (e.g. regression coefficient) AND variation (e.g. standard deviation) or associated estimates of uncertainty (e.g. confidence intervals)
☐	☒	For null hypothesis testing, the test statistic (e.g. F, t, r) with confidence intervals, effect sizes, degrees of freedom and P value noted<br>*Give P values as exact values whenever suitable.*
☒	☐	For Bayesian analysis, information on the choice of priors and Markov chain Monte Carlo settings
☐	☒	For hierarchical and complex designs, identification of the appropriate level for tests and full reporting of outcomes
☐	☒	Estimates of effect sizes (e.g. Cohen's d, Pearson's r), indicating how they were calculated

Our web collection on statistics for biologists contains articles on many of the points above.

Software and code

Policy information about availability of computer code

Data collection	Statistical and molecular data analyses were carried out using the “R” statistical computing environment (‘R’ statistical software version 3.4.4, RRID:SCR_001905) and associated modules from Bioconductor in the Linux operating system (Ubuntu, 16.04.4).
Data analysis	Custom R codes were used for all of the statistical analyses. Source codes are available upon request (email the corresponding author).

For manuscripts utilizing custom algorithms or software that are central to the research but not yet described in published literature, software must be made available to editors and reviewers. We strongly encourage code deposition in a community repository (e.g. GitHub). See the Nature Research guidelines for submitting code & software for further information.

Data

Policy information about availability of data

All manuscripts must include a data availability statement. This statement should provide the following information, where applicable:

- Accession codes, unique identifiers, or web links for publicly available datasets
- A list of figures that have associated raw data
- A description of any restrictions on data availability

The exome mutations, somatic copy number alterations and gene expression data that support the findings are available in figshare with the identifier DOI: 10.6084/m9.figshare.14710425.

Field-specific reporting

Please select the one below that is the best fit for your research. If you are not sure, read the appropriate sections before making your selection.

- [x] Life sciences
- [ ] Behavioural & social sciences
- [ ] Ecological, evolutionary & environmental sciences

For a reference copy of the document with all sections, see nature.com/documents/nr-reporting-summary-flat.pdf

Life sciences study design

All studies must disclose on these points even when the disclosure is negative.

Sample size	The 274 cell lines used for the in vitro drug screening were selected based on their origin and growth properties. The aim was to cover the principle cancer (sub)types and to have enough cell lines per cancer (sub)type to allow statistics. Of which, 237 have full profiles and were used in the statistical analysis
Data exclusions	No data, cell line or molecular dataset were excluded from the analyses.
Replication	In vitro drug screening was performed in singlicate.
Randomization	not applicable
Blinding	not applicable

Reporting for specific materials, systems and methods

We require information from authors about some types of materials, experimental systems and methods used in many studies. Here, indicate whether each material, system or method listed is relevant to your study. If you are not sure if a list item applies to your research, read the appropriate section before selecting a response.

Materials & experimental systems

n/a	Involved in the study	
x		Antibodies
	x	Eukaryotic cell lines
x		Palaeontology and archaeology
x		Animals and other organisms
x		Human research participants
x		Clinical data
x		Dual use research of concern

Methods

n/a	Involved in the study	
x		ChIP-seq
x		Flow cytometry
x		MRI-based neuroimaging

Eukaryotic cell lines

Policy information about cell lines

Cell line source(s)	Two hundred and seventy-four CLs from the Oncotest GmbH repository (Freiburg Germany, since 2015 Charles River Discovery Research Services (DRS) Germany GmbH) were used in the study. Cell lines were either developed by Oncotest GmbH from patient-derived xenografts or obtained from DSMZ, CLS, NCI, ATCC, ECACC, KCLB, JCRB, Public Health England.
Authentication	Tumour cell line authenticity was confirmed by short tandem repeat analysis at DSMZ (Braunschweig, Germany).
Mycoplasma contamination	The cell lines were tested for mycoplasma by PCR and were all mycoplasma free.
Commonly misidentified lines (See ICLAC register)	The cell lines SNB-19 and TE671 were included in the cell line panel used for the in vitro drug sensitivity screening. The molecular data of the cell line SNB-19 was also included in the bioinformatics analyses. The SNB-19 cell line is described to be a U-251 MG derivative, while the TE671 is a RD (rhabdomyosarcoma) derivative.
